# Discorhabdin N, a South African Natural Compound, for Hsp72 and Hsc70 Allosteric Modulation: Combined Study of Molecular Modeling and Dynamic Residue Network Analysis

**DOI:** 10.3390/molecules24010188

**Published:** 2019-01-06

**Authors:** Arnold Amusengeri, Özlem Tastan Bishop

**Affiliations:** Research Unit in Bioinformatics (RUBi), Department of Biochemistry and Microbiology, Rhodes University, Grahamstown 6140, South Africa; g16a7782@campus.ru.ac.za

**Keywords:** human cancers, South African natural compounds, heat shock proteins, molecular dynamics, allosteric drugs

## Abstract

The human heat shock proteins (Hsps), predominantly Hsp72 and Hsp90, have been strongly implicated in various critical stages of oncogenesis and progression of human cancers. While drug development has extensively focused on Hsp90 as a potential anticancer target, much less effort has been put against Hsp72. This work investigated the therapeutic potential of Hsp72 and its constitutive isoform, Hsc70, via in silico-based screening against the South African Natural Compounds Database (SANCDB). A comparative modeling approach was used to obtain nearly full-length 3D structures of the closed conformation of Hsp72 and Hsc70 proteins. Molecular docking of SANCDB compounds identified one potential allosteric modulator, Discorhabdin N, binding to the allosteric β substrate binding domain (SBDβ) back pocket, with good binding affinities in both cases. This allosteric region was identified in one of our previous studies. Subsequent all-atom molecular dynamics simulations and free energy calculations exhibited promising protein–ligand association characteristics, indicative of strong binding qualities. Further, we utilised dynamic residue network analysis (DRN) to highlight protein regions actively involved in cross-domain communication. Most residues identified agreed with known allosteric signal regulators from literature, and were further investigated for the purpose of deducing meaningful insights into the allosteric modulation properties of Discorhabdin N.

## 1. Introduction

Rapid acquisition of somatic mutations by human cancers in reprisal to drug pressure is a major setback encountered in cancer treatment [1]. Despite great advances by experts, the development of effective antineoplastic drugs remains frustratingly elusive. Often, tumorigenic cells induce several pro-survival systems [2,3] via activating a number of pathways and over-expressing highly conserved molecular chaperones—heat shock proteins (Hsps)—in order to augment drug resistance. Within human Hsp molecular chaperones, the family of 70 kDa heat shock proteins (Hsp70) plays a central role in promoting cancer resistance and longevity. Scientific reports have well demonstrated the over-reliance of multiple tumour types on Hsp70s for a wide scope of cancer-relevant biological activities [4], besides assisting in routine cellular polypeptide folding processes [5]. Under unstressed conditions, normal cells ubiquitously express the constitutive isoform, Hsc70, and in minimal quantities, Hsp72 [6]. Usually, Hsp72 is upregulated in response to stress stimuli, and is thought to enhance heat shock tolerance and restore systemic balance [6]. Following extrinsic and intrinsic stressors, including cytotoxic drugs, UV irradiation, low pH [7], limited glucose [8], and hypoxia [9], tumour cells express uncommonly high levels of both Hsp72 and Hsc70 constitutively [10]. Increased expression of Hsp72 and Hsc70 has been implicated in tumour pro-survival through modulation of critical cell-signalling pathways that control cell fate. For instance, accumulation of Hsp72 was found to suppress oncogene-induced senescence mechanisms [11] and block stress-initiated apoptotic pathways [10], among other complex biochemical processes. Moreover, there is evidence that elevated expression of Hsp72 correlates with poor disease prognosis and poor clinical outcomes from chemotherapy [12].

Hsp72 involvement in diverse roles predisposes it as an ideal therapeutic avenue to treatment of a broad spectrum of tumour types. The combination of Hsp70 isoforms and the 90 kDa heat shock protein (Hsp90) is complementary in orchestrating protein folding and translocation [13]. Hsp72 and Hsc70 are required for delivery of “client proteins” onto Hsp90 machinery, as well as to participate in the assembly of Hsp90 directed heterocomplexes [13,14]. Numerous reports have strongly implicated Hsp72 and Hsc70 in reduced sensitivity of Hsp90 to anti-cancer inhibitors [15,16]. Recently, it was observed that silencing both Hsp72 and Hsc70 was lethal on cancer cells but not on untransformed epithelial cells, and more strikingly, it improved Hsp90 sensitivity to anticancer drugs [16]. To this end, drug combinations or use of multi-target directed drugs seems the most suitable strategy to achieve robust inhibitory effects.

**Figure 1:**
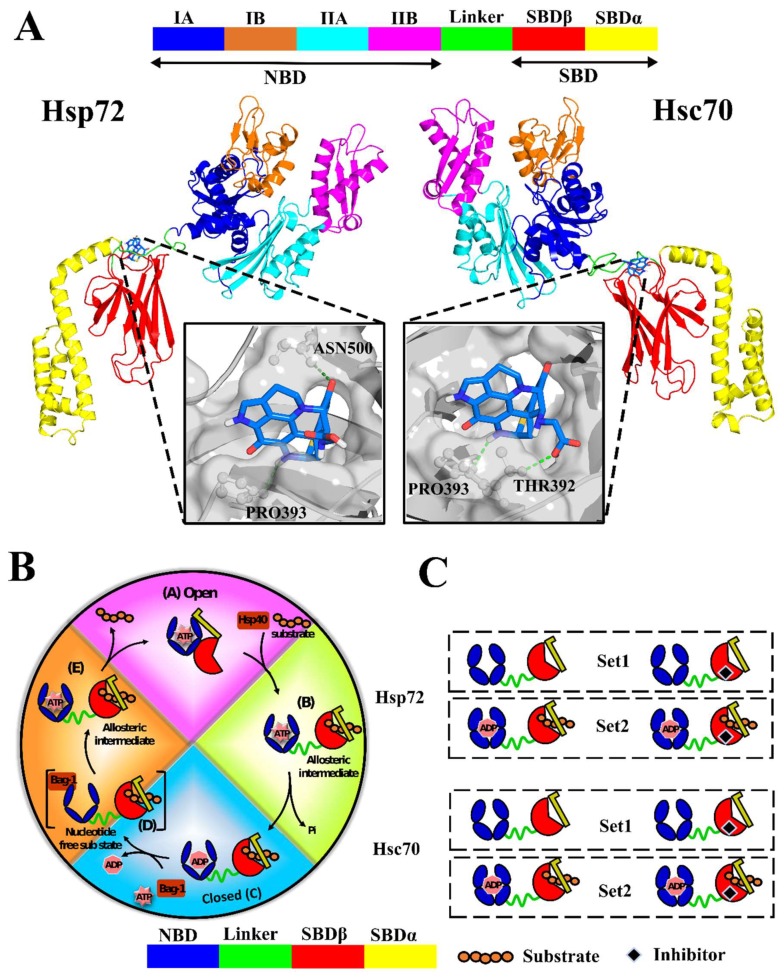
(**A**) Cartoon representation of Hsp72 and Hsc70 3D structures calculated via homology modelling. The nearly full-length closed confirmation structures of Hsp72 and Hsc70 were modelled from *Escherichia coli* DnaK PDB entry: 2KHO. Predicted binding mode of SANC00132 docked to the allosteric target site, the SBDβ back pocket, is shown in sticks. Polar contacts are displayed as green dashes while interacting residues are shown in ball and stick. (**B**) Canonical nucleotide dependent cycle of Hsp70. (**C**) Schematic illustration of the experimental approach. Set1 comprises the simulation of SANC00132-free and SANC00132-bound systems only. Set2 simulations are for SANC00132-free and SANC00132-bound systems complexed with endogenous modulators (ADP and peptide substrate). Concisely, each protein was subjected to both sets of experiments.

Availability of structural data has advanced the understanding of the family of Hsp70 proteins′ architecture and functional mechanisms. Structurally, Hsp70s have two main allosterically-coordinated domains; the nucleotide binding domain (NBD), and substrate binding domain (SBD), connected by a short flexible linker (Figure 1A) [17] The NBD is made up of two adjoining lobes (lobe I and lobe II) constructing a deep nucleotide binding groove and connected at the base. Each lobe consists of two subdomains (IA, IIA, IB, and IIB) [18,19]. The SBD is composed of a β subdomain (SBDβ) and an α-helical subdomain (SBDα) that operates as a lid, which covers the substrate binding pocket and entraps the bound peptide [20].

Based on exposure of the substrate binding pocket to client polypeptides, Hsp70 occupies two functionally important conformations that can be defined as “open” (substrate-free), and “closed” (substrate-bound) [18]. The closed state is typically elongated, ADP-bound, and possesses high affinity for peptide substrates. The open conformation is compact, ATP-bound, and is characterised by high rates of association or dissociation of the substrate [21,22]. To function, Hsp70 transitions between both conformations (open and closed) in a cyclic fashion initiated by nucleotide hydrolysis [21,22,23]. Association with co-chaperone Hsp40 and the nucleotide exchange factor (Bag-1) regulates substrate binding and release and nucleotide exchange activities [19,21,24]. Figure 1B gives a schematic illustration of the nucleotide driven Hsp70 cycle: (A) Binding of the substrate to the accessible substrate binding pocket and interaction of the open state with Hsp40 initiates allosteric signals and stimulates ATPase activity [17,18]. Allosteric signals prompt spontaneous structural rearrangements involving domain decoupling and closure of SBDα, stabilising the bound peptide in a partially closed allosteric intermediate [18,22,25]. (B) Hsp40 interaction, besides substrate binding activities accelerates ATP hydrolysis, promoting the rapid switch to the closed state [17,26]. Dynamics of the flexible interdomain linker, involving alternative contacts with either the NBD or SBD, is thought to further modulate ATPase activity, and importantly mediate cross-domain allostery [27]. (C) Interaction with a nucleotide exchange factor (Bag-1), which recognizes ADP bound Hsp70, disrupts the conformational equilibrium by mechanically opening lobes IB and IIB, facilitating the release of ADP [28,29,30,31,32]. (D) The result is a transient NEF stabilized nucleotide-free sub state of comparable interdomain orientation as the preceding closed conformation [17,32,33,34]. (E) Bag-1 allows fast ATP exchange and subsequent transition to the open state [34]—ATP binding triggers allosteric signals that prompt domain repositioning and partial docking of the SBD to the NBD (allosteric intermediate). It also disfavours SBDβ–SBDα interaction, facilitating the displacement of the substrate for another cycle of substrate binding [21,35].

Several studies have been performed to design potential inhibitors against Hsp72, including the identification of 2-phenylethynesulfonamide (PES) [36], 15-deoxyspergualin (DSG) [37], natural products Oridonin [38] and Novolactone [39], and an irreversible inhibitor 8-*N*-benzyladenosine [40]. The hypothesis of dual-targeting Hsp72 and Hsc70 was recently explored by Jones and co-workers [41], where truncated NBD protein structures were screened and fragment hits based on aminoquinazoline scaffolds identified. However, targeting the NBD is complicated by the fact that the domain is highly conserved [6], is almost always occupied by ADP and ATP, and that most amino acids lining the ADP and ATP binding pocket are hydrophilic, hence “difficult” drug binders [42,43]. Furthermore, as the NBD and SBD are allosterically coordinated, targeting a single domain in an isolated manner would not present the behaviour of the entire protein under the selected conditions. Therefore, this work focuses on inhibitor design against the entire protein, while specifically aiming to target the relatively less conserved SBD and looking at potential allosteric sites, rather than substrate binding areas that can be defined as the active site of the domain. Based on a recent review [25], targeting allosteric sites of this poorly explored domain is likely to provide selective and less toxic small compound modulators of Hsp70. Previously, the compound Triphenyl(phenylethynyl)phosphonium (PET-16) was described to specifically bind the SBD and inhibit ADP-bound Hsp70 [44,45].

In our previous study, we used all-atom molecular dynamics (MD) simulations coupled with perturbation response scanning (PRS) to determine allosteric hotspots that may be implicated in modulating conformational dynamics in *E. coli* Hsp70 and DnaK [18]. Due to the high conservation properties of Hsp70s, findings on DnaK have often been interchangeable with Hsp70s of other species. Hence, we extrapolated the information for the potential allosteric hotspot sites to human Hsp72 and Hsc70 proteins. Here, we propose that force perturbations at these hotspot sites are a natural consequence of binding forces caused by protein–protein or protein–ligand interactions; and that these sites may be suitable allosteric drug target candidates. Recently, this hypothesis was, for the first time, successfully applied to allosteric modulator design against human Hsp90α protein [46].

In light of the above, our study focused on identification of potential allosteric modulators from the South African Natural Database (SANCDB) [47]. Towards this goal, accurate near full-length 3D models of both proteins in the closed conformation were calculated using homology modelling techniques. Discorhabdin N (SANCDB id: SANC00132) is identified as an allosteric regulator against both Hsc70 and Hsp72. Discorhabdin N is a pyrroloiminoquinone-type marine alkaloid commonly isolated from Latrunculid sponges [48,49]. The compound was previously shown to possess in vitro anticancer activity against HCT116 human colon cancer cells [49]. Moreover, its analogs have shown significant in vivo activity in preclinical tests against prostate tumor [50].

In a neutral solvated system, all-atom molecular dynamics (MD) simulations of Hsp72 and Hsc70 complexed with SANC00132 in the presence and absence of endogenous modulators were performed to determine protein–ligand behaviour. It was observed that the presence of SANC00132 in Hsp72 allows steadfast binding of ADP to its native binding site, triggers the advancement of the SBD towards the NBD, and locks both domains creating a unique rigid closed conformation. The dynamic behaviour of ADP and SANC00132-bound proteins were visualised using VMD, interdomain movements were tracked by measuring distances between the centre of mass of both domains, and general conformational rearrangements, besides protein flexibility, were explored using essential dynamics.

In our analysis, we proposed an integrated approach of incorporating structure-based drug design methods with dynamic residue network (DRN), relying on the rationale that the steady interaction of potential regulators with critical allosteric signal mediators would greatly increase the likelihood of conception of allosteric inhibition. Accordingly, key communication centres that could be responsible for regulation of cross-domain allosteric signals were identified using DRN analysis. Existing protein–ligand interactions were evaluated for their contribution to the total binding free energy, as well as their potential to effectively cause allosteric inhibition from interactions with identified communication hubs. It was established that SANC00132 possesses the ability of allosterically modulating both proteins by disruption of cross-domain information flow. Its association with Hsp72 was more prominent.

Overall, we report a natural compound with high binding affinity for an allosteric site located in the SBD of anti-cancer drug targets, Hsp72 and Hsc70. Our findings provide novel insights regarding the modulation of conformational dynamics in the closed conformation of both proteins in response to the bound ligand and allow us to discuss the possible allosteric mechanism.

## 2. Results and Discussion

### 2.1. Accurate Nearly Full-Length Protein Structures Are Calculated via Homology Modeling

In the absence of experimentally resolved crystal structures, homology modelling is exceptionally useful in the prediction of accurate 3D structures. Initially a suitable template was identified using PRIMO [24]. PRIMO implements both HHsearch [51] and protein BLAST [52] options along with various alignment tools for improved accuracy. Considered during template selection was completeness of the structure, besides sequence identity and secondary structure similarity. PDB ID 2KHO possessed an overall well-ordered structure with 80% query coverage, 48% sequence identity and 0.779 secondary structure similarity. Target sequences were aligned to the template in a multiple sequence alignment and models built and validated. Initially, generated models were evaluated for their global structure quality and ranked using normalized z-DOPE, a distance-dependent statistical potential [53]. Typically, low z-DOPE values correspond to native-like structures. Top models of Hsp72 and Hsc70 recorded −0.51 and −0.56 z-DOPE scores respectively, indicating reasonably accurate structures. Assessment of stereochemical parameters using PROCHECK server provided a Ramachandran plot showing the distribution of torsion angles [54]. 93.7% and 94.1% respectively of all residues possessed torsion angles in the most favoured region characteristic of proteins with high geometric accuracy (Table S1, Figure S2). Comparative evaluation of how close the proteins’ 3D structures agreed with their own sequence (1D) showed that at least 80% of all amino acids scored 0.2 or more in the 3D/1D profile assessment indicating high 3D/1D compatibility [55] (Table S1). Further, comparisons against high resolution protein chains of similar size available in the PDB using ProSA web-server [56] yielded Z-scores within the range of experimentally determined proteins suggesting high structural closeness (native-like). Also, the resultant values were largely negative (−10.98 and −11.07 respectively) indicating proper protein geometries (Table S1, Figure S1). Altogether, these results show that accurate and high-quality structures were built.

### 2.2. A Natural Compound Is Identified via Molecular Docking for the Selected Allosteric Site

Previously, allosteric hotspots that may be implicated in modulating conformational dynamics in *E. coli* Hsp70, DnaK [18] were identified. Here, the information was extrapolated to the human Hsp72 and Hsc70 protein structures with the idea that these sites may be suitable allosteric drug target candidates for cancer research. One of these regions, at the back pocket of SBDβ was also identified as allosteric druggable pocket by Rodina et al. [42]. In this study, we identified a druglike natural compound targeting this pocket of both proteins.

AutoDock Vina [57] utilises a fast-stochastic search method to exploit degrees of freedom assigned to a flexible ligand within a defined search space. Re-docking of ADP to 3ATV reproduced a binding pose comparable to its native orientation when superimposed, indicating that our docking protocol was justifiable (data not shown). High throughput virtual screening was performed on Hsp72 and Hsc70 as described in the Materials and Methods section. Predicted ligand poses were ranked by Vina [57] and XScore [58] scoring tools starting with the energetically most favourable pose. In practice, a consensus from both functions could improve the enrichment of true positives [59]. SANC00132, identified among the top twenty hits preferably binding at the allosteric target site of both proteins, yielded low binding energies of −6.9 Kcal/mol and −7.1 Kcal/mol (Vina scores), and 5.51 pKd and 5.74 pKd (XScore scores) in Hsp72 and Hsc70 in that manner (Table S2). Post-docking analyses revealed common molecular interactions including ARG504, PRO393 and ALA392THR (Figure 1A and Figure S2, Table S3). ASN500 (Hsp72) and GLY479 (Hsc70) were other interacting residues. In this paper all residue numbering corresponds to *E. coli* DnaK sequence PDB ID: 2KHO (UniProtKB–P0A6Y8), our reference sequence/structure, unless specified otherwise. Table S3 presents equivalent residue numbering in respective proteins. Hsp72 residues PRO393 and ASN500 formed two hydrogen bonds with the ligand, whereas Hsc70 had one with THR392. These results provide promising initial binding poses; hence a potential hit—Discorhabdin N.

Prediction of oral bioavailability properties enhances the isolation of active compounds with viable scaffolds. Ideally, desirable drug candidates should comply with at least four of Lipinski RO5 test parameters [60]. SANC00132 passed 4 test parameters and displayed sufficient acceptable drug likeness properties (Table S4). Based on these results, the ligand possesses drug-like properties and is likely suitable for oral formulations. PAINS motif filters, however, revealed a Quinone A substructure ingrained in SANC00132, an indication that the compound could possibly be a promiscuous binder. On the other hand, identification of PAINS motif should not be sufficient enough to discard the compound from being potential drug candidate in an early stage as there are several FDA approved small molecule drugs containing PAINS features. For instance, Eltrombopag is used for thrombocytopenia, or low platelet count [61]. Additionally, Doxorubicin and Mitoxanthrone, possessing the Quinone substructure [62,63], are used as anticancer chemotherapy drugs [63].

### 2.3. Effects of Ligand Binding on Global Protein Motions are Observed

**Figure 2:**
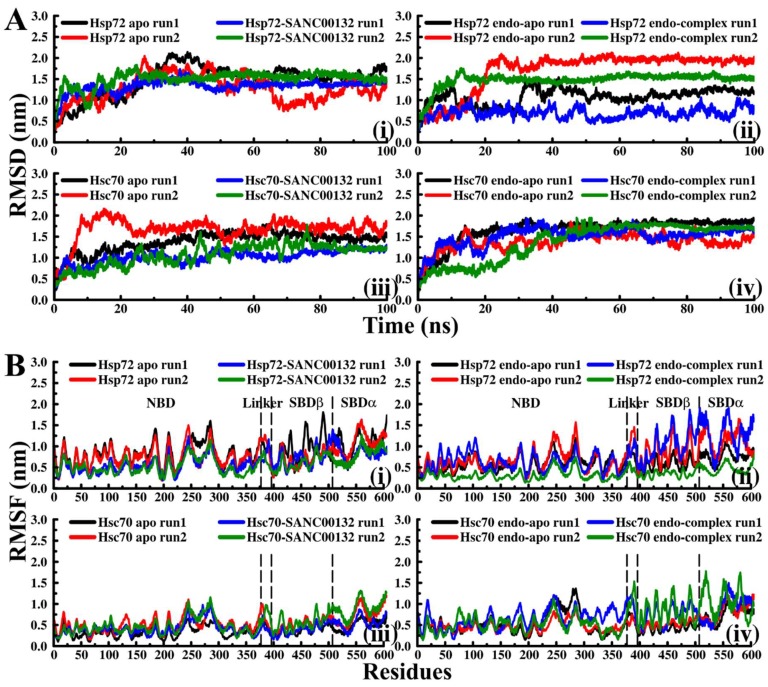
(**A**) RMSD evolution of back-bone atoms. (**B**) Average RMSF of residues computed from C_α_ atoms. (**i**) Hsp72 Set1, (**ii**) Hsp72 Set2, (**iii**) Hsc70 Set1, (**iv**) Hsc70 Set2. Colour key: Black and red: SANC00132-free proteins. Blue and green: SANC00132-bound proteins.

**Figure 3:**
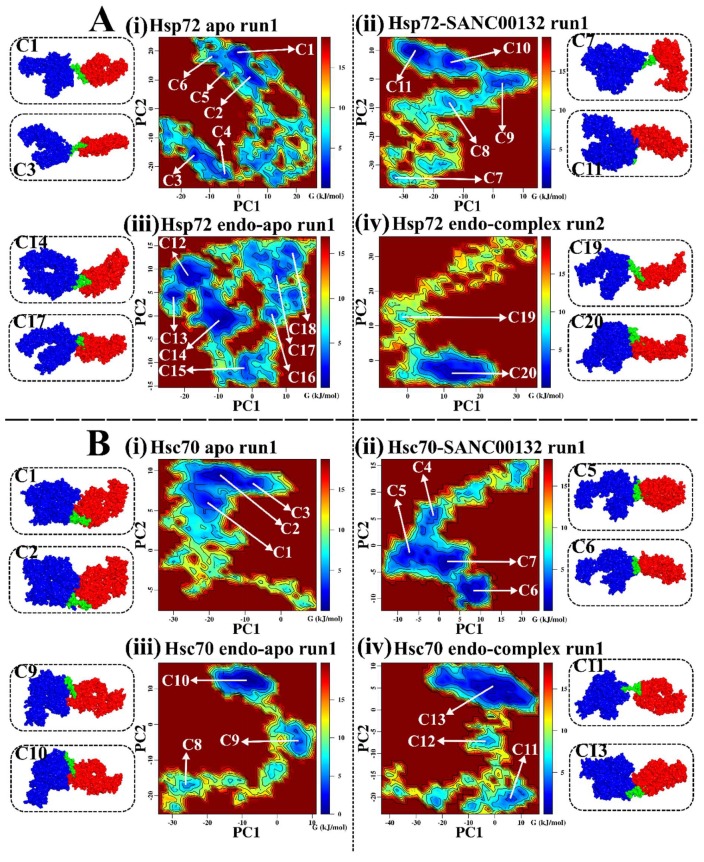
(**A**,**B**) 2D projections of free energy landscape along the first and second principal components. Comparisons between SANC00132-free and SANC00132-bound systems. Surfaces are coloured based on Gibbs free energy levels from maroon (high energy/maxima) to blue (low energy/minima). Each contour represents an increase of the free energy of 1 kJ·mol^−1^. Conformations sampled are labelled from C1–C20 (Hsp72) and C1–C11 (Hsc70), respectively. Snapshots of representative conformations occupying important free energy wells (minima) are shown as surfaces and coloured domain-wise: Blue: NBD, Green: Linker, Red: SBD. **A**: Hsp72: (**i**) Hsp72 apo Run1, (**ii**) Hsp72-SANC00132 Run1, (**iii**) Hsp72 endo-apo Run1, (**iv**) Hsp72 endo-complex Run2. **B**: Hsc70: (**i**) Hsc70 apo Run1, (**ii**) Hsc70-SANC00132 Run1, (**iii**) Hsc70 endo-apo Run1, (**iv**) Hsc70 endo-complex Run2. Duplicate trajectory results are shown in the Supplementary Data (Figure S5).

Two sets of dynamic simulations (Set1 and Set2) were performed on Hsp72 and Hsc70 as described before (Figure 1B). Initially the potential, kinetic and total energy of all systems were checked to establish the accuracy of experimental parameters (data not shown). Calculation of RMSD evolution offers insights on conformational adaptability and the overall stability of protein–ligand complexes. RMSD values of ligand-bound systems of both proteins mostly converged early and evenly indicating fairly stable systems (Figure 2A, Table S5). Also, RMSDs of ligand-free systems, predominantly Hsc70 systems, converged well. Variations in RMSD values of Hsp72 apo (run1 and run2), Hsp72 endo-complex run1, Hsc70 apo run1, and Hsc70 endo-apo run2 indicated general flexibility attributes of the full-length Hsp70 closed conformation structure, and particularly demonstrated the hinging dynamics of the flexible interdomain linker; relative to the NBD, the SBD swivels freely during simulation. RMSD jumped around 30–40 ns in Hsp72 endo-apo systems, which coincided with spontaneous dissociation of ADP into solution from the NBD (Video S1). Based on Hsp70s allosteric cycle, the succeeding conformational transition entails participation of NEF in indirect distortion of the nucleotide binding cleft, consequently enhancing ADP release [30,32,34]. ADP dissociation noted in Hsp72 endo-apo systems could be attributed to the significant structural flexibility and conformational plasticity of ligand-free Hsp72 compared to ligand-free Hsc70, as determined by essential dynamics calculations (see Figure 3 and Figure S5). Both the nucleotide and bound peptide substrate are important for stabilization of the closed conformation [18,45,64]. The removal of ADP destabilizes the complex, yielding numerous ensembles of metastable nucleotide-free conformers, as shown in Figure 3A(iii). In contrast, the nucleotide stays bound to Hsp72 endo-complex systems (Video S2), suggesting that the binding of SANC00132 possibly imposes a restraining effect on ADP and could consequently affect the efficacy of its release.

To monitor per residue fluctuations, RMSF was calculated based on Cα atoms. Overall, residues of ligand-bound Hsp72 structures display lower RMS fluctuation values compared to similar residues of ligand-free structures (Figure 2B). Particularly, the SBDβ displays minimal RMS fluctuations, suggesting favourable protein-inhibitor association. Residues of inhibitor-bound Hsc70 structures, however, show slightly higher RMS fluctuation values, indicating that SANC00132 binding generates mild structural agitation.

Based on C_α_ atom positions, radius of gyration (Rg) was computed against time to examine the radial adjustment (expansion or compaction) of protein structures with respect to their centre of mass. It was clear that ligand-bound Hsp72 attained a more compact structure, suggesting a tight receptor grip on SANC00132 (Figure S3). Altogether, the Rg values decreased initially and eventually plateaued at a value of around ~3.4 nm for the last 40 ns period. Besides Hsc70-SANC00132 run1, Rg differences recorded between ligand-free and ligand-bound Hsc70 systems were minimal, indicating that SANC00132 binding marginally alters the spatial packing of the residues.

#### Discorhabdin N Locks Hsp72 Domains Establishing a Rigid Closed Conformation

Trajectories were visually inspected using VMD. Strikingly, binding of SANC00132 to the allosteric region triggers structural rearrangements in Hsp72 (Video S3 and Video S4). The SBD appears to be towed by the conserved interdomain linker towards the NBD in a flip and drag motion, before settling into an NBD-SANC00132-SBD sandwich. Repositioning of the SBD was clearly recognizable in Hsp72-SANC00132 complexes (Video S4). Accordingly, interdomain advances were tracked in all systems by measuring the dynamic distance between the centre of mass of the SBD and that of the NBD. Given that duplicate MD runs may not always be in agreement, ligand-bound Hsp72 systems (Hsp72-SANC00132 Run1, Hsp72 endo-complex Run1, and Hsp72 endo-complex Run2) largely registered an initial drop in the distance up to a steady value (~5.5 nm) that was maintained for the rest of the simulation period. However, Hsc70 systems (both ligand-free and ligand-bound), besides Hsc70-SANC00132 Run1, display indistinct differences (Figure S3). These results support Rg findings and the plots, as well display a comparable pattern. It is evident that ligand-bound Hsp72 adopts a compact equilibrium conformation by locking both domains.

To account for collective structural motions and prevailed conformational sampling during simulation, essential dynamics were performed on trajectories. The atomic covariance and subsequent eigenvalue and eigenvector matrices were built from coordinates of C_α_ atoms and diagonalized using *gmx covar* and *gmx anaeg* tools in that manner. First and second principal components (PC1 and PC2) captured dominant protein motions as depicted in Table S6 by the percentage variance contribution. Respective eigenvectors (out of a total of 1818) were used to generate scatter plots (Figure S4). Compared to clusters of ligand-free systems, ligand-bound Hsp72 yielded well defined clusters, indicative of a smaller conformational space sampled during simulation, while Hsc70 systems exhibited paltry differences. To clearly demonstrate SANC00132 effects on conformational heterogeneity at 300 K, associated free energy landscapes (FEL) were determined as a function of the top two principal components (PC1 and PC2). FEL can be used to adequately describe conformational redistributions induced by binding events [65,66]. Figure 3 displays the relative conformational changes of Hsp72 and Hsc70. In reference to this figure, we could identify that ligand-free Hsp72 (both apo and endo-apo) evolves through many different intermediate conformations linked by low lying energy barriers. The protein structures populate 6 (C1–C6) and 7 (C12–C18) main sub-states for apo and endo-apo systems, respectively. This indicates that Hsp72 switches through diverse ensembles of flexible conformations during 100 ns simulation. Ligand-bound Hsp72 is characterized by 5 (C7–C11) and 2 (C19–C20) energy minima wells for the Hsp72-SANC00132 complex and Hsp72 endo-complex, respectively. Interestingly, compared to ligand-free energy surfaces, there is a considerable population shift towards a single-well landscape (towards C11 and C20), suggesting global conformational switching to a single metastable state. Respective representative structures further depict that the protein evolves into a stable non-native fold and this could signify ligand-induced modulation of the folding process. Ligand-free Hsc70 samples 3 conformational subspaces in both apo (C1–C3) and endo-apo (C8–C10) systems. This is indicative of a more relaxed and fairly inflexible protein system. Ligand-bound Hsc70 visits 4 (C4–C7) and 3 (C11–C13) conformational subspaces for the Hsc70-SANC00132 complex and Hsc70 endo-complex, in that manner. Besides the Hsc70-SANC00132 complex, which depicted a reversed population shift relative to the apo system, energy surfaces of both ligand-free and ligand-bound systems display a nearly similar distribution of energy minima basins. These results lead us to the conclusion that SANC00132 strongly modulates Hsp72 protein conformations, and moderately modulates Hsc70 conformations, and thus could regulate the proteins′ function.

Trace values (sum of 1818 eigenvalues of each protein) of diagonalized covariance matrices represent the overall amplitude of atomic vibrations and altogether depict the underlying structural flexibility. The calculated average standard deviation of trace values between duplicate runs (Run1 and Run2) was found to be 426.86 nm^2^. The Hsp72 endo-complex registered a large variance between Run1 and Run2 trace values (var = 1522120.50), as shown in Table S6. Overall, ligand-bound Hsp72 systems (considering Hsp72-SANC00132 Run1, Hsp72-SANC00132 Run2, and Hsp72 endo-complex Run2 trajectories) recorded lower trace values than ligand-free systems (Table S6), suggesting decreased protein flexibility and hence more stable complexes. Ligand-bound Hsc70 consistently registered slightly higher trace values in both runs than ligand-free systems, indicating minimally enhanced protein flexibility due to ligand binding. Thus, from the above results, it was concluded that ligand-bound Hsp72 complexes were more stable.

### 2.4. Evaluation of Binding Affinity Suggests Favorable Protein–Ligand Associations

**Figure 4:**
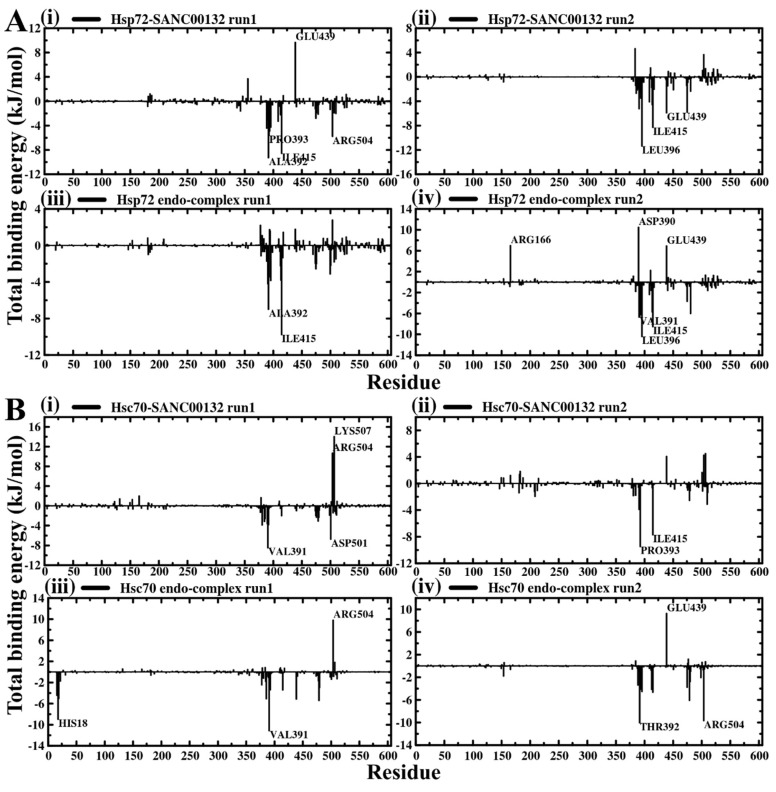
Bar plots showing per residue contribution to total binding free energy. (**A**) Hsp72 SANC00132-bound complexes. (**B**) Hsc70 SANC00132-bound complexes. All residues contributing more than +/−5 kJ·mol^−1^ are listed in Table S7.

Binding free energy estimates have become important in drug discovery since they describe the robustness of non-bonded molecular associations. The more negative the resultant value, the more potent the association between the ligand and protein is, and vice versa. Using the MM-PBSA method [67,68], binding free energy (ΔG_binding_) was computed as the difference between the free energy of the protein and that of SANC00132 on the la36st 15 ns trajectory snapshots, sampled at 10 ps time intervals. Although both complexes yield low binding free energies, demonstrating favourable protein–ligand interactions, ligand-bound Hsp72 was found, on average, to possess a slightly lower value (by about ~ −10 kJ·mol^−1^) than Hsc70, indicating enhanced binding affinity. In both cases, the van der Waals energy term (Δ*E*_vdW_) largely contributed to negative energetic values (Table 1). To get detailed information on binding, the total binding free energy was decomposed on a per residue basis. Residue clusters within ligand binding sites of both proteins, including LEU389-LEU396, ILE409-ILE415, PHE473-LEU481 for Hsp72 (LEU395-LEU401, ILE414-ILE420, PHE478-LEU486 complete Hsp72 sequence equivalent) and ASP378-LEU396, ILE415, ASP474-ILE480, THR499-GLU511 for Hsc70 (ASP383-LEU401, ILE420, ASP479-ILE485, THR504-GLU516 complete Hsc70 sequence equivalent), made substantial contributions to the total binding free energies (Figure 4). Table S7 gives a comprehensive report of all residues identified. Of note, the ligand interactions with Hsp72 GLU439 (3.12 kJ·mol^−1^), and Hsc70 ARG504 (3.77 kJ·mol^−1^) and LYS507 (5.29 kJ·mol^−1^) yielded unfavourably large positive binding free energy values on average.

We have shown that SANC00132 binds favourably to both Hsc70 and Hsp72 and stably locks the functional domains of the latter. To elaborate further, underlying non-covalent bonds and existing molecular interactions were calculated and visually inspected using *gmx hbond* [69], DS Visualizer [70], and LigPlot+ tools [71]. Hydrogen bond participation in binding influences the overall ligand binding affinity. Its implications for protein–ligand complex stability are immense. Time dependent calculations on hydrogen bonds formed between proteins and SANC00132 during simulation show that, on average, 4 hydrogen bonds consistently formed between Hsp72 and SANC00132, and at least 2 were always present (Figure S6). Hsc70 complexes form inconsistent numbers of hydrogen bonds with SANC00132. Overall, the variation is between 0 and 8, which decreases significantly in Hsc70 endo-complexes to the range of 0–3 after 50 ns (Figure S6). Descriptively, hydrogen bonds mostly formed with LEU394, SER413, and GLU439 of Hsp72 (LEU399, SER418, GLU444 complete Hsp72 sequence equivalent) and GLN384 of Hsc70 (GLN389 complete Hsc70 sequence equivalent) (Figure S10 and Figure S11). Identified residues compare well with major binding free energy contributors. Based on these results, it can be concluded that hydrogen bond formation is a dominant driving force to the stabilization of SANC00132-bound Hsp72.

### 2.5. Dynamic Residue Network Analysis (DRN) Reveals Critical Communication Centers

**Figure 5:**
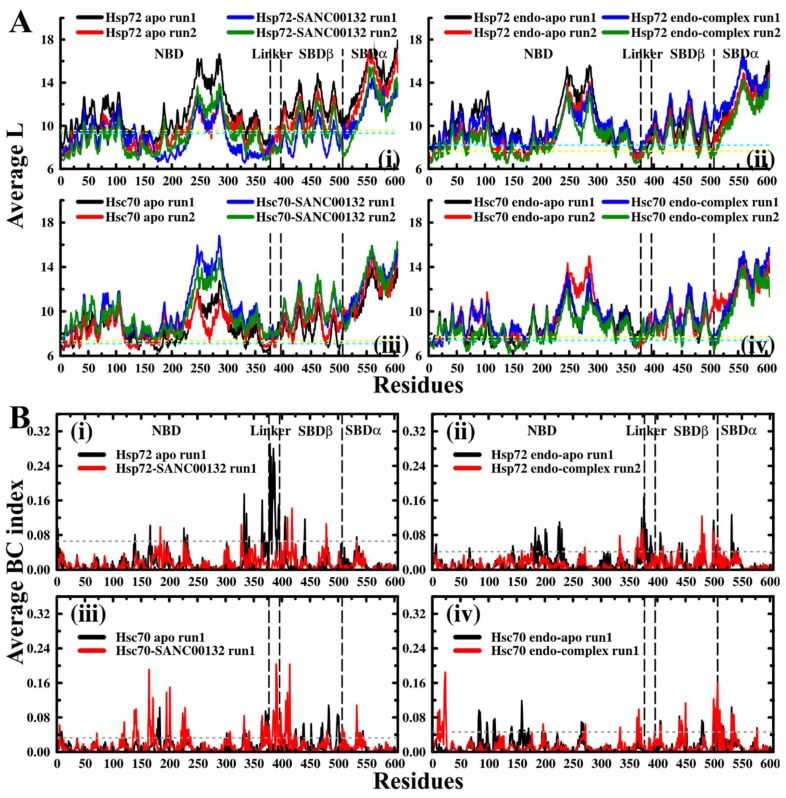
Contact network analysis: (**A**) Average shortest path (L) results. Colour key: Black and red: SANC00132-free, blue and green: SANC00132-bound. The upper threshold values of 9.31 (Hsp72 apo run1), 8.17 (Hsp72 apo run2), 8.20 (Hsp72 endo-apo run1), 7.65 (Hsp72 endo-apo run2), 7.09 (Hsc70 apo run1), 7.35 (Hsc70 apo run2), 7.40 (Hsc70 endo-apo run1), and 7.69 (Hsc70 endo-apo run2) are indicated by the dotted lines. Colour code: Cyan: run1, yellow: run2. (**B**) Betweenness centrality (BC) results. Colour key: Black: SANC00132-free, red: SANC00132-bound. The lower threshold values of 0.07 (Hsp72 apo run1), 0.04 (Hsp72 endo-apo run1), 0.03 (Hsc70 apo run1), and 0.03 (Hsc70 apo run1) are indicated by the grey dotted lines. Results of duplicate trajectories are shown in Figure S7.

**Figure 6:**
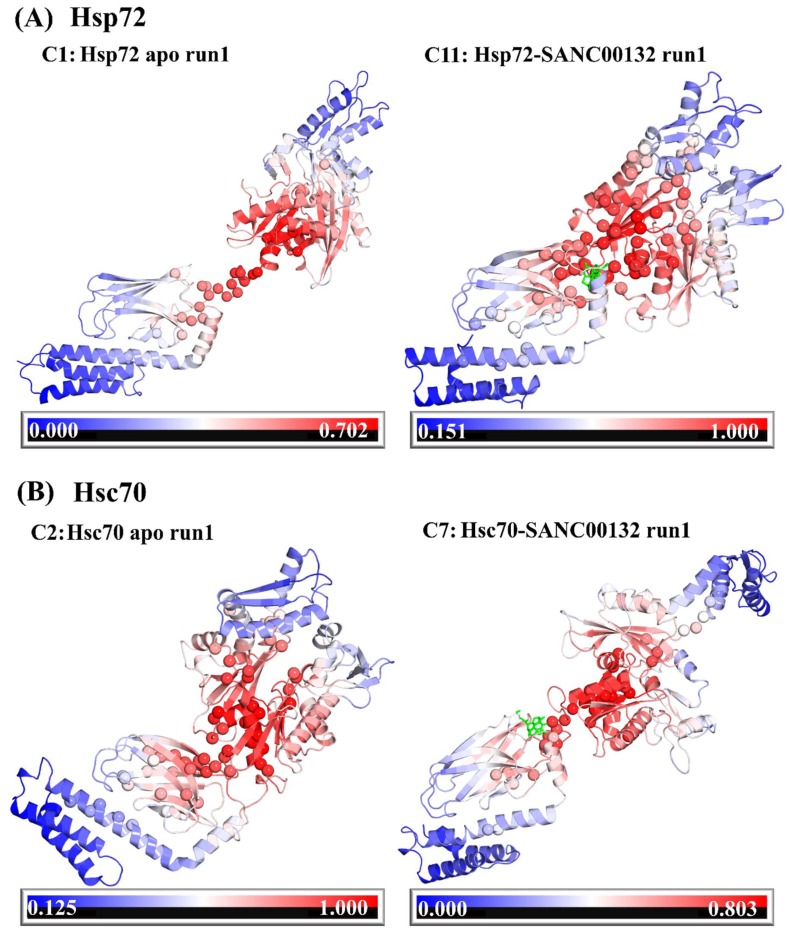
Structural mapping of average L and average BC on SANC00132-free and SANC00132-bound Hsp72 and Hsc70 representative structures. Structures were retrieved from the largest clusters occupying important free-energy basins conformers (**A**: C1 (73 ns), C11 (82 ns), and **B**: C2 (56 ns), C7 (96 ns) in relation to Figure 3). Structures were coloured based on normalised average L values from red (lowest value; highly accessible) to blue (highest value; limited accessibility). Residues bearing the highest BC indices are indicated as spheres. First, both Hsp72 and Hsc70 images depict the global effects of ligand binding on structure compaction as described by Rg and interdomain distance results. Binding of SANC00132 to Hsp72 promotes intraprotein reorganisation from a loosely packed (more elongated) ligand-free 3D structure to a more compact complex. The opposite holds true for Hsc70. Second, it is evident that residue communities with significant average L and average BC values are populated at the interdomain linker, nucleotide, and substrate binding sites. SANC00132 is represented in sticks and coloured green.

Backbone connectivity impacts the efficiency of signal transmission and hence the rate of conformational change of allosteric proteins [72,73]. The average shortest path (L) highlights the mean topological spread of all residues from a given residue by considering the shortest paths to every other residue constituting the network. L portrays the sociability of a residue in a communication network [73]. The lower the L value, the higher the availability of the residue for signal transduction. Dips of average L plots represent residues possessing low average L values. Since the values yielded were more spread out, a value of one standard deviation away from the mean was used to identify these residues from ligand-free proteins, which ideally characterize native residue connectivity (Table 2, Table S8A,B). We found that the distribution of residues having low average L values was generally similar between Hsc70 and Hsp72. Consistent in both proteins, residue communities located within ADP and substrate binding sites (SBDβ), including residues 1–17, 119–127, 135–146, 163–180, 331–337, 476–481, and 500–504, remarkably recorded low average L values (Figure 5A and Figure 6). These results indicate that the identified sites are placed in intimate proximity to most amino acids in the network and are therefore readily accessible for communication. Other than known active sites, residues in significant regions, including the stretch between the α-helical NBD C-terminal through the interdomain linker (residues 359–384, 397–398), yielded low average L values. Remarkably, some of the residues and residue clusters identified at the SBD are in agreement with previous findings by Penkler et al. from perturbation response scanning (PRS) experiments on full-length *E. coli* DnaK proteins [18] as well as Verkhivker and co-workers from contact network analysis on truncated substrate bound and unbound DnaK and Hsp72 proteins [45].

**Figure 7:**
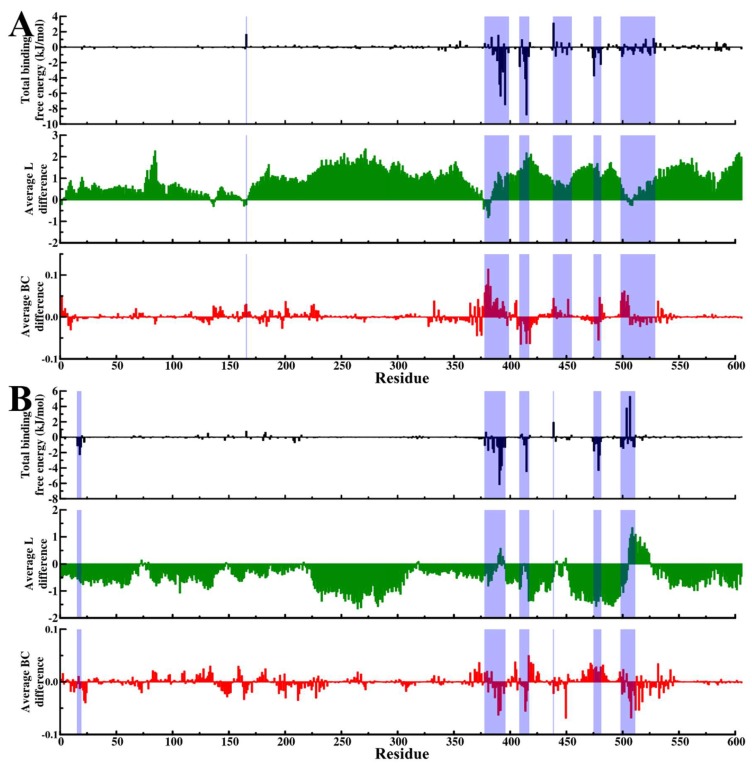
Effects of ligand binding on residue accessibility and centrality. Graphical mapping of structural regions making significant contribution to total binding free energy (shaded blue) onto plots of average L difference (green) and average BC difference (red). Change in residue accessibility (average L difference) and residue centrality (average BC difference) were obtained from calculations of SANC00132-free less SANC00132-bound values. For each metric, per residue means across both sets (set1 and set2) of experiments was initially calculated. (**A**) Hsp72, (**B**) Hsc70.

Based on average L plots (Figure 5A), we observed that generally, the network topology in both ligand-free and ligand-bound systems was preserved. To clearly reveal the effects of ligand binding on residue accessibility, the difference in average L values between SANC00132-bound and SANC00132-free trajectories was calculated (ligand-free less ligand-bound). Residues 3–133, 139–161, 167–376, 384–504, 506, and 510–606 for Hsp72 and 1–71, 77–147, 150–216, 218–317, 320–389, 393, 396–412, 414–440, 443–447, 449, 451–505, and 526–606 for Hsc70 registered negative and positive changes in Average L values in that manner. The above range of residues constitute nearly 95% of the full-length sequence of Hsp72 or Hsc70, signifying collective global shifts. Concisely, Hps72 recorded a general decrease (negative change) in average L values, while Hsc70 registered an increase (positive change) (Figure 7A,B middle panels). These results could be easily interpreted as enhanced residue accessibility for Hsp72 and vice-versa for Hsc70 because of ligand binding. Markedly, residue clusters corresponding to regions participating in ligand binding (residues contributing significantly to total binding free energy), including residues 372–384, 394–395, 400, 439–454, and 501–515 for Hsp72 and 387–396, 412–415, 441–450, and 506–525 for Hsc70, largely yielded either minimal or positive changes in Average L values for Hsp72, and either minimal or negative changes in Average L values for Hsc70. These observations suggest ligand-induced restraints to global shifts in Average L values in these sections. According to recent studies on residue interaction network behaviour by Santoni and co-workers [74], the group reported the existence of a strong correlation between “global” average shortest path and structure compaction (Rg). The general increase in Average L values in Hsp72 and decrease in Hsc70 observed here follow similar qualitative tendencies observed in radius of gyration results, and therefore hint at a possible correlation between average L and Rg metrics. For instance, shortened path length indicated by the global negative vertical shift of ligand-bound Hsp72 curves (Figure 5A(i)) compare to condensed protein mass distribution results of the same (Figure S3A(i)). In summary, while the average shortest path “scaled” globally because of ligand binding in both Hsp72 and Hsc70, ligand-binding regions opposed these concerted changes. Ultimately, the implication is reduced residue accessibility for Hsp72 and vice-versa for Hsc70 in ligand-binding sections.

In a recent study, an inverse correlation between L and BC was shown [75]. Here, to complement average L findings, BC indices of residues were calculated. Essentially, betweenness index denotes the magnitude of participation of a node in the shortest paths between all residue pairs, and thus its centrality. Peaks of average BC plots represent residues possessing high betweenness indices. Generally, the distribution of significant peaks in SANC00132-free profiles (both apo and endo-apo) was relatively similar. Since the values were more centred around the mean compared to average L values, a value of two standard deviations was used to identify these residues from ligand-free systems (Table 3, Table S9A,B). Overall, we found that residues stretching between the NBD α-helical C-terminal and the interdomain linker (ALA372-SER395 for Hsp72: ALA377-SER400 complete Hsp72 sequence equivalent, VAL370-LEU387 for Hsc70: VAL375-LEU392 complete Hsc70 sequence equivalent) clearly registered the highest betweenness values (Figure 5B and Figure 6). These results support published literature sources heavily implicating the interdomain linker to regulation of allosteric transition through adjudication of cross-domain allosteric signals [18,27]. Remarkably, residues identified here (Table 3), also agree well with PRS results on *E.coli* DnaK [18] opening transition experiments.

Next, we looked at SANC00132-complexed systems. As expected, presence of SANC00132 altered the betweenness profiles of both proteins. Similar observations were made in a previous study on DnaK and Hsp72 in complex with PET-16 [45]. To comprehensively show the effects of SANC00132 binding on residue centrality, the difference between SANC00132-free and SANC00132-bound trajectories was calculated (ligand-free less ligand-bound). Residues 369–395, 405–410, 413–420, 438–441, 476–482, and 499–509 for Hsp72 and 21–24, 133–136, 144–142, 164–167, 195–200, 369–375, 390–395, 405–407, 414–421, 450, 470–484, and 500–512 for Hsc70 yielded either negative or positive substantial changes in centrality index on average (Figure 7A,B lower panels). Generally, most residue directly involved in SANC00132 binding displayed distinct fluctuations in betweenness centrality indices.

Even though the representative complex (Hsp72-SANC00132 run1) depicts increased numbers of highly connected residues appropriately due to decreased threshold value applied (0.04 relative to 0.07 for Hsp72 apo run1) (Figure 6), BC profiles of ligand-bound Hsp72 displayed an overall decline in betweenness indices of significant peaks (Figure 5B, Figure 7A). Notably, the NBD α-helical C-terminal and inter-domain linker registered a dramatic drop in betweenness indices, suggesting that allosteric signalling is not any more mediated by native critical communication hubs. Likewise, while Hsc70-SANC00132 run1 (representative complex) showed reduced numbers of highly connected residues appropriately due to increased threshold value (0.05 relative to 0.03 for Hsc70 apo run1) (Figure 6), ligand-bound Hsc70 yielded an increased number of irregular betweenness peaks in general (Figure 5B, Figure 7B). Emergence of uncommon connections implies disintegrated and a distorted information flow network and may signify future unpredicted biological effects.

#### Average L Compares to Average BC and Residue Fluctuations (RMSF)

We observed similar trends between per residue RMS fluctuation and average L profiles. Also, key communication residues identified from BC and L calculations nearly overlap. We therefore tested for any correlation between average BC, average L, and RMSF. Recently, the possible correlation between these three metrics, for the first time, for flexible and rigid protein systems (Hsp90 open and closed confirmations, respectively) was investigated by Penkler et al. [75]. It was shown that there is a good correlation, especially in the rigid protein systems.

**Figure 8:**
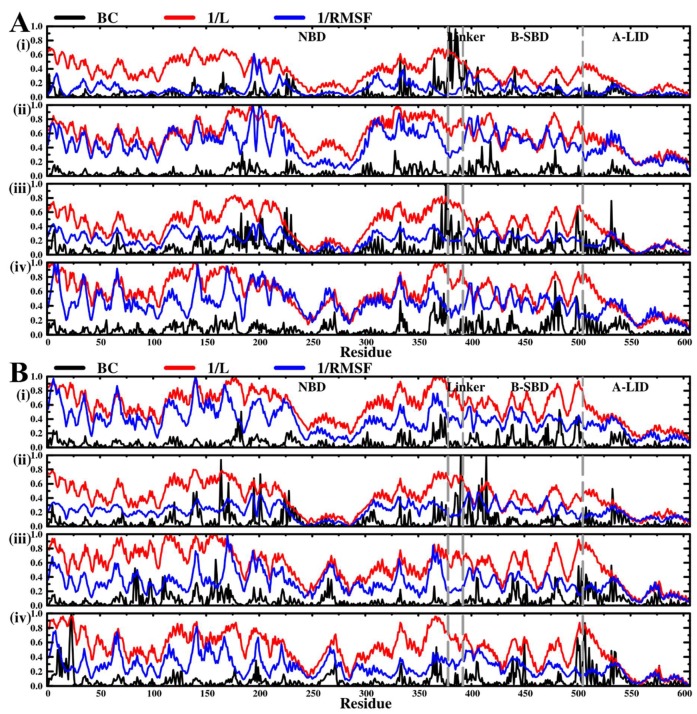
Graphical illustration of correlation between 1/RMSF, 1/Average L, and Average BC. (**A**) Hsp72 (**B**) Hsc70. Pairwise comparisons between 1/BC and 1/L as well as comparisons from duplicate trajectories are found in Supplementary Data (Figure S16 and Figure S17). **A**: (**i**) Hsp72 apo run1, (**ii**) Hsp72-SANC00132 complex run1, (**iii**) Hsp72 endo-apo run1, (**iv**) Hsp72 endo-complex run2. **B**: (**i**) Hsc70 apo run1, (**ii**) Hsc70-SANC00132 complex run1, (**iii**) Hsc70 endo-apo run1, (**iv**) Hsc70 endo-complex run1.

Figure 8, Figure S8 and Figure S9 give a graphical overview of identified relationships. RMSF computed over the last 15 ns recorded marginal improvement in the correlation coefficient by nearly 0.09 against both average BC and average L. Overall weak correlation observed between BC and both L^−1^ and RMSF^−1^ could possibly be attributed to the highly dynamic nature of the nearly full-length structures along with enhanced structural flexibility observed during simulation.

Using the pairwise Pearson’s correlation coefficient, RMS fluctuation values calculated over a 100 ns period and over the last 15 ns were separately compared against BC and L (Table 4). Similar calculations were also performed domain-wise. We report that average BC is weakly correlated to the inverse of RMSF (RMSF^−1^), regardless of the sampling period, (r = 0.16 (100 ns), r = 0.25 (last 15 ns). While there is moderate linear relationship between average BC and the inverse of average L, r = 0.45, a strong degree of correlation exists between RMSF and average L, (r = 0.61 (100 ns), r = 0.69 (last 15 ns). The latter result agrees with a recent pairwise comparison reported by Penkler and colleagues [75]. Domain-wise calculations display only slight improvements in correlation values, as shown in Table S10.

### 2.6. Challenging the Capability of Discorhabdin N to Regulate Cross-Domain Communication

In analysing molecular interactions, we argue that protein–ligand non-bonded contacts involving amino acids crucial for allosteric communication enhance the likelihood of allosteric modulation by the ligand through disruption of the native information flow network. Besides site-directed mutagenesis studies, the use of a dynamic residue interaction network could be applied in uncovering signal-sensitive contacts (“trigger” spots) necessary for inception of ligand-induced allosteric regulation signals. Consistent with this thought, interacting residues were compared against key communication mediation hubs identified from average L and average BC calculations. Further comparisons were made against residues identified from published literature sources (Table S11). Among residues participating in protein–ligand interactions, eight of Hsp72 and seven of Hsc70 possessed high centrality indices and matched residues previously implicated in allosteric control of protein functions (Table S11). Remarkably, most Hsp72 and Hsc70 interacting residues cluster along the SBD N-terminal and the C-terminal tail of the conserved interdomain linker instrumental for mediating allosteric cross-domain communication [27].

Specifically, interactions with hinge residue GLN384 of Hsc70 (LYS387 [27] equivalent of DnaK) and residues ALA392 (Hsp72) and VAL391 (Hsc70) corresponding to the conserved LDVT C-terminal motif of the linker [27] may provide convincing explanation why SANC00132 binding has immense implications on conformational dynamics of both proteins. Constraining the activity of hinge bending GLN384 could engender unusual rigidity of the linker, thereby restricting succeeding interdomain coupling events. ALA392 and VAL391 interactions could impair the efficiency of interdomain cross-talk, thus disrupting allostery-guided transition from the closed to the allosteric intermediate states. Apart from compromising signal propagation [18], stable interactions with residues PRO393 (Hsp72 and Hsc70), LEU394 (Hsp72), and LEU396 (Hsp72) proximal to the conserved linker may incidentally influence the kinetics of the linker; it is likely that ligand interactions with multiple residues within this region, (SBDβ N-terminal) as identified in Hsp72, dominate the observed complacent manipulation of the protein by the ligand. Notably, interactions with LEU394, LEU396, ILE409, and LYS410 of Hsp72 (Table S11 displays complete sequence equivalent) were absent in Hsc70. These unique interactions could be examined in order to delineate prospective pharmacological qualities ((inverse) agonism or antagonism) of SANC00132 on either protein. Moreover, they could be directly implicated in the observed modest variation in allosteric modulation propensities between Hsp72 and Hsc70. Indeed, point mutation experiments involving LEU394 (LEU397) showed deleterious effects on DnaK activity in vivo [76]. Qi and co-workers reported that the residue was indispensable in facilitating interdomain coupling by participating in the formation of SBDβ–NBD contacts [76]. Thus, based on the above findings, it can be proposed that SANC00132 holds the potential of directly interfering with interdomain communication in both proteins and has high chances of allosterically impeding Hsp72 functioning via interaction with LEU394 (LEU399 complete Hsp72 sequence equivalent).

On the other hand, interdomain locking actions described earlier (Figure S3B, Video S2) and systemic evidence of relative redistribution of conformations (population shifts) (Figure 3, Figure S5) point at two prospective regulatory mechanisms: First, that SANC00132 could accelerate the formation and stabilization of the NBD–SBDβ interface in Hsp72, hence favouring succeeding domain docking events crucial for appropriate allosteric regulation of the chaperone function [17]. Second, that SANC00132 could stabilize Hsp72 or Hsc70 in the closed state, allowing extended periods of ADP/substrate association. Longer ADP/substrate association times could interrupt normal progression of the Hsp70 cycle and consequently influence product (folded polypeptides) turnover rates.

**Figure 9:**
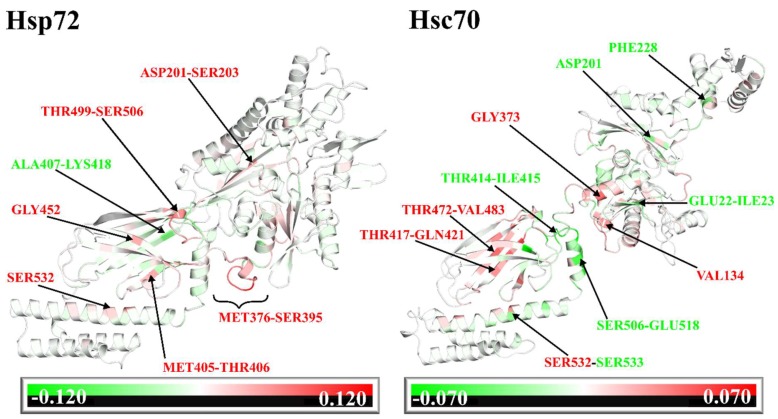
Structural mapping of residues showing significant changes in centrality index on snapshots of Hsp72-SANC00132 run1 and Hsc70-SANC00132 run1. Regions were coloured from green (negative changes or increased residue centrality) to red (positive changes or decreased residue centrality).

Comparing SANC00132-induced network changes with that induced by PET-16 at the SBD of DnaK [45], we find considerable differences in adjustment patterns of residue centrality. While PET-16 largely increased centrality of binding site residues in DnaK, SANC00132 induces reduced centrality, particularly on residues 405, 406, 499-506, and 452 of Hsp72 and residues 417-421 and 472-483 of Hsc70 (Figure 9, Figure S12). Other notable inclusions, overriding qualitative measures (increase or decrease), were LEU394, LYS410, and ILE415 (Hsp72) and VAL391, THR392, and ILE415 (Hsc70) directly participating in SANC00132 binding interactions (Table S12). These results indicate that the two ligands could engender opposing signals that differentially regulate chaperone functions. Besides binding site residues, distant effects, specifically on SER532 in the SBDα of both proteins (SER537 complete Hsp72/Hsc70 sequence equivalent) were noticeable. SER532 borders the conserved VAL531 (VAL536) important for formation of SBDβ–SBDα interactions that practically stabilize the shut lid [77]. These findings suggest that SANC00132 allosteric modulation may involve adjustment of SBDβ–SBDα contacts, thus indirectly affecting the substrate affinity. In regard to the full-length structure, both Hsc70 and Hsp72 demonstrated relatively consistent patterns of residue clusters that yielded substantial changes in centrality index (Figure S12). Residues were arranged in three major regions between positions 1–24 (lobe IA), 133–237 (lobes IA, IIA and IIB), and 328–543 (long stretch of residues from lobe IIA through the interdomain linker, the SBDβ, and the SBDα N-terminal). Changes in centrality indices of residues located at lobes IA, IIA, and IIB of the NBD suggest that SANC00132 binding may not only influence the dynamics of the bound peptide, but also the nucleotide (ADP). By drawing these observations together, it can be reasoned that the changes, or no change, in residue centrality because of ligand binding may be mandatory for stabilization of the complex in the adopted conformation that favours cooperativity between modulator binding sites and distant protein regions (active sites). Moreover, these changes could be well fashioned to yield receptor-specific organization of the residue interaction network that ultimately determines enhanced (agonism) or reduced (antagonism) modes of target activity.

## 3. Materials and Methods

**Figure 10:**
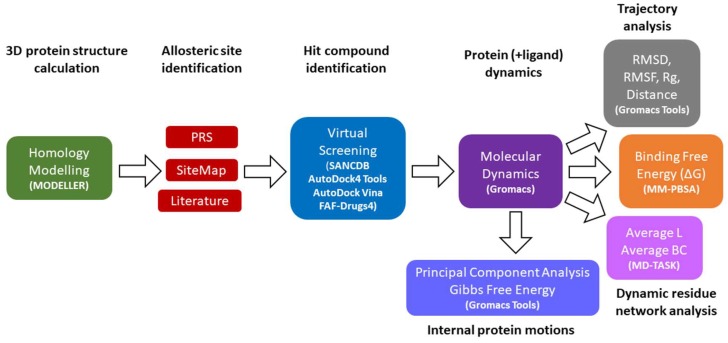
A workflow illustration of methodology. The figure schematically describes the main steps performed and tools employed as indicated in brackets in this work.

In this study, we integrated high-throughput virtual screening with rigorous all-atom molecular dynamics simulations and dynamic residue network analysis (DRN) techniques, to investigate allosteric regulation capabilities of a potential small compound modulator on nearly full-length human Hsp72 and Hsc70 models (Figure 10).

### 3.1. Sequence Retrieval and Homology Modelling

Structure-guided drug discovery efforts for human Hsc70/Hsp72 have been limited by lack of available structural data. Here, we employ comparative modelling techniques to obtain nearly full-length 3D structures. Complete human Hsc70 and Hsp72 sequences with accession numbers AAK17898.1 and NP_005337.2, respectively, were retrieved from the NCBI database. For each sequence, template structure identification was performed using the PRIMO web server [24]. A nearly full-length closed conformation crystal structure of DnaK, PDB entry 2KHO, was selected, and the coordinate file was retrieved from PDB. Alignment output from PRIMO was used to generate pir files for homology modelling. Model calculation was performed for residues between 6 and 606 for both protein sequences, with very slow refinement using MODELLER version 9.16 [78]. The C-terminal end (606–641) was excluded from modelling due to un-availability of templates. Hundred models were built for each protein totalling to 200 models. The normalised z-DOPE (Discrete Optimized Protein Energy) score was used to initially rank predicted models [53]. The top three models per protein proceeded to local quality evaluation using PROCHECK [79], ProSA[56], and Verify3D tools [55]. The most accurate model of each protein was used for molecular docking studies.

### 3.2. Virtual Screening and Druglikeness Prediction

SBD of both proteins was objectively targeted with 623 geometrically refined and minimised structures of natural compounds retrieved from the South African Natural Compounds Database (SANCDB) web server [47]. To predict the preferred allosteric binding sites of compounds, virtual screening was performed on the entire protein surface of apo proteins (we refer to the nucleotide/substrate void protein structures as “apo”) using AutoDock Vina [57]. A rigid receptor, flexible ligand approach was used. Default parameters for number of ligand torsion bonds were assigned. Receptor and ligand preparation were performed using AutoDockTools4 [80]. Initially, docking validation was conducted to assess the ability of AutoDock Vina to reproduce correct binding poses. Co-crystallised ADP was re-docked to the crystal structure (PDB ID: 3ATV) and its spatial position and orientation compared to that of the native complex for the validation purpose. As the proteins are elongated in their closed confirmation, it was not feasible to set up only one grid box for blind docking experiments. Thus, docking of compounds to the NBD and SBD involved definition of two grid box search spaces of dimension sizes x = 48Å, y = 62Å, and z = 80Å, and x = 36Å, y = 48Å, and z = 75Å, concentrating on each domain separately and respectively with some overlap in the linker area. Ligands were centred at x = 0.0, y=0.0 anf z = 0.1 for NBD and x = 0.0, y = −9.7, and z = −63.2 for SBD, and a search exhaustiveness value of 512 was employed for both domains. Docking parameters used for NBD matched those used in the docking validation process. For each protein–ligand complex, ten docked ligand poses were generated and ranked using Vina score. To increase the confidence in detection of true positives, ligand poses were independently rescored using XScore tool [58] and ranked. Identification of potential hits from the top compounds having low binding energies was construed as a consensus of both Vina and XScore results. Ligands binding at the allosteric SBDβ back pocket identified by Rodina et al. [42] were objectively isolated using in-house Python scripts. Besides assessing acceptability properties of the top twenty compounds for oral formulation, Lipinski RO5 [60] was implemented in order to accommodate the small pocket size characteristics of the binding site. Prediction of drug-like properties, along with other molecular descriptors, were performed on the FAF-Drugs4 web server [61]. Favourable compounds were visually inspected for non-bonded interactions using Discovery Studio Visualizer 4 (DS Visualizer) [70] and LigPlot+ [71]. To identify possible PAINS-like structural motifs (Pan Assay Interference Compounds), the ZINC15 database [81] filter was employed.

### 3.3. Molecular Dynamics Studies

To explore the innate protein dynamics of Hsc70 and Hsp72 systems, molecular dynamics (MD) simulations were performed on supercomputers at the Centre for High Performance Computing (CHPC) in Cape Town, using GROMACS 5.1.2 package [69]. An all atom AMBER03 force field [82] was implemented. The study design involved two sets of simulation for each protein (Figure 1C): The first simulation set (Set1) included SANC00132-free and SANC00132-bound protein systems named as Hsp72 apo, Hsp72-SANC00132 complex and Hsc70 apo, Hsc70-SANC00132 complex, accordingly. The second set (Set2) involved simulations of SANC00132-free and SANC00132-bound proteins in complex with endogenous modulators (ADP and peptide substrate) and were called Hsp72 endo-apo, Hsp72 endo-complex and Hsc70 endo-apo, Hsc70 endo-complex. Throughout the text, we will commonly refer to SANC00132-free systems as “ligand-free” and SANC00132-bound as “ligand-bound”. Collectively, each protein was subjected to both sets of experiments (Figure 1C). Duplicate all-atom MD simulations with different random seeds were performed for all systems. Overall, 16 MD simulations were done. All experiments were carried out under similar conditions of 300K temperature, 1 bar pressure, and a neutral pH. In the preparation of endo-apo and endo-complex systems, endogenous modulators (ADP and peptide substrate) were obtained by superimposition of the target proteins with PDB ID: 1DKZ and PDB ID: 3ATV and extrapolation of the respective atomic coordinates. Topology files for proteins (Hsc70 and Hsp72) and ligands (ADP, SANC00132, and peptide substrate) were generated using Gromacs *pdb2gmx* tool [69] and ACPYPE software [83], respectively. Initially, ligands were protonated using DS Visualizer. A triclinic periodic box with a minimum distance of 2.0 Å from the protein edge was defined and filled with water molecules using the explicit simple point charge (SPC126) water model. The systems were electro-neutralised by replacing a proportion of water molecules with 0.15 M NA^+^ and Cl^−^ counter-ions. While applying the steepest descent method, the systems were subjected to a maximum of 50,000 steps of energy minimisation without position restraints until it converged with a force less or equal to 1000 kJ/mol/nm. 100,000 steps of equilibration under NVT (canonical) and NPT (isothermal-isobaric) conditions succeeded, respectively, with position restraints on all heavy atoms. NVT equilibration utilised the modified Berendsen thermostat for temperature coupling. The reference temperature was set to 300 K with a coupling time constant of 0.1 ps. Parrinello-Rahman barostat was used for NPT equilibration. Additionally, 1 bar pressure and a coupling time constant of 2.0 ps were set. To separately and accurately couple the systems’ components, the Leap-frog integrator was applied. Lennard Jones potential was implemented in the calculation of Van der Waals interactions. A cut-off distance of 1.4 nm was used. Likewise, evaluation of long-range electrostatic interactions was performed using the Particle Mesh Ewald method with the coulomb cut-off distance fixed at 1.4 nm. Bonds on all atoms were constrained using Lincs algorithm. Finally, all systems were subjected to 100 ns production runs with 0.002 ps timestep and periodic boundary conditions in all directions. Coordinates were stored in every 2 ps.

### 3.4. Trajectory Analysis

The root mean square deviation (RMSD), root mean square fluctuation (RMSF), radius of gyration (Rg), and number of hydrogen bonds (H) were calculated using Gromacs tools [69] *gmx rms*, *gmx rmsf*, *gmx gyrate,* and *gmx hbond,* respectively. Distances between the centre of mass of the NBD and that of the SBD were calculated using the *gmx dist* tool. Initially, trajectories were visually inspected using visual molecular dynamic software (VMD) [84]. To identify sustained protein–ligand interactions, geometric clustering of ligand RMSDs over the last 15 ns of convergence was done using *gmx cluster* tool. The gromos method and 0.025 nm RMSD grouping cut off were used. Complexes were rendered using DS Visualizer and LigPlot+.

Description of internal protein motions was accomplished using principal component analysis (PCA). This entailed (1) Construction of the covariance matrix (**C**) from (x,y,z) coordinate positions of Cα atoms as representatives of residues (**N**), generating a large matrix of dimension *3N × 3N*. *gmx covar* tool was used; (2) diagonalization of **C** to obtain eigenvectors sorted based on associated eigenvalues using *gmx anaeg*; and (3) construction of Gibbs free energy profiles as a function of the top two eigenvectors (PC1 and PC2) using *gmx sham*, *xpm2txt.py*, and *sham.pl* tools. Data handling and visualisation of plots were done using R and Excel spreadsheet.

### 3.5. Binding Free Energy Calculations

Free energy calculations were performed using the molecular mechanics Poisson-Boltzmann surface area (MM-PBSA) method [67]. MM-PBSA calculations are fast and have effectively been applied in several scientific works [85,86]. The *g_mmpbsa* tool [68] was used. Solvation free energy (polar and non-polar solvation energies) and molecular mechanics energy (Van der Waals and electrostatics) were computed using this tool. Essentially, the endpoint states (bound and unbound) are considered in MM-PBSA calculations. Here, trajectory snapshots of the last 15 ns were sampled at time step intervals of 10 ps. To gain further insights into binding, the total binding free energy was decomposed on a per residue basis.

### 3.6. Dynamic Residue Network Analysis

Construction of contact network graphs and subsequent analyses were performed using MD-TASK tool [87]. MD-TASK implements NetworkX Python libraries to build and compare residue communication graphs. Residues, in this case C_β_ atoms (C_α_ for glycine), are nodes and edges connecting nodes represent non-bonded interactions or links. A minimum interaction cut off distance of 6.7 Å was used to define existing links. The dynamic residue network (DRN) is collated from the residue interaction network (RIN) calculations of trajectory snapshots sampled over a stipulated simulation period. Here, frames traversing the last 15 ns were sampled at time step intervals of 10 ps. MD-TASK calculates matrices of residue betweenness centrality (BC) and residue accessibility (L) indices, and further computes the running averages thereof. Average L and average BC options were used to analyse trajectories. To calculate L, the path bearing the minimum number of links connecting a reference residue *n*_0_ to another residue *n*_1_ is initially identified from all other possible paths. Successively, similar paths from *n*_0_ to every one of the residues (*n*_2_, *n*_3_, *n*_4_…) in the network are also determined. All shortest paths from *n*_0_ are summed up and divided by the total number of residues, less one. The resultant value represents the average length of shortest paths from residue *n*_0_ (L). BC describes how often a node is visited by all other nodes along their shortest paths from all others. Average L and average BC values were obtained from calculations of the mean of L and BC values accrued from individual frames spanning a specified simulation period, in this case the last 15 ns. Further, pairwise Pearson correlation coefficient values were computed between average L, average BC, and RMSF. To identify these relations and visualise results, at first values of each of the above metrics were separately normalised between 0 and 1 using the following equation.

(1) $$Z_{A}=\frac{A-min\left(A,B\right)}{max\left(A,B\right)-min\left(A,B\right)}$$

(2) $$Z_{B}=\frac{B-min\left(A,B\right)}{max\left(A,B\right)-min\left(A,B\right)}$$

where *A* = ligand-free matrix (e.g., Hsp72 endo-apo), and *B* = ligand-bound matrix (e.g., Hsp72 endo-complex). *Z_A_* and *Z_B_* are the normalised data for ligand-free and ligand-bound matrices respectively.

## 4. Conclusions

Targeting allosteric regions for inhibitor design is gaining importance, as the inhibitors for the allosteric target sites can be very specific. In our previous study, we identified allosteric hotspots that may be implicated in modulating conformational dynamics in *E. coli* Hsp70, DnaK [18]. Here, we extrapolated the information for the potential allosteric hotspot sites to human Hsp72 and Hsc70 proteins, with the idea that these sites may be suitable allosteric drug target candidates, as demonstrated in a recent study [46]. Hsp72 and Hsc70 are important anti-cancer drug targets, and their inhibition can be helpful in salvaging biochemical pathways that determine cell fate.

This study identified Discorhabdin N (SANDDB id: SANC00132) as a potential allosteric modulator for both human Hsp72 and Hsc70 proteins through molecular docking and dynamics studies. Protein-inhibitor associations were comprehensively analysed through duplicate 100 ns MD simulations using different random seeds in the explicit water model. It was observed that binding of Discorhabdin N to Hsp72 advances structural reorganisations that culminate in the formation of a unique NBD- Discorhabdin N-SBD lock. Also, it was noted that its association with Hsp72 could impede ADP dissociation from the NBD. Compared to Hsc70, the Hsp72 protein is more susceptible to modulation by Discorhabdin N. Not only did the modulator bind more stably to Hsp72, but also formed inflexible and compact protein–ligand complexes. Binding free energy calculations yielded favourable binding affinities of Discorhabdin N to both Hsc70 and Hsp72. The higher number of non-covalent hydrogen bonds formed with Hsp72 confirmed a robust protein–ligand association. Using DRN analysis, key communication centres were revealed in both proteins based on average L and betweenness centrality (BC) calculations. We have reported a strong correlation between average L and RMS fluctuation. Comparisons between protein–ligand interacting residues and identified critical communication residues established that Discorhabdin N is likely to disrupt cross-domain communication in both proteins. Ligand interactions with LEU394 (LEU399 complete Hsp72 sequence equivalent), hinge residue GLN384 of Hsc70 (GLN389 complete Hsc70 sequence equivalent), and residues corresponding to the conserved LDVT C-terminal motif of the linker ALA392 for Hsp72, and VAL391 for Hsc70 (ALA397 and VAL396 complete sequence equivalents respectively) will be an important factor considered during hit optimisation and could serve as a starting point towards understanding the allosteric modulation mechanism of Discorhabdin N on Hsp72 and Hsc70. Additionally, residues exhibiting significant changes in residue interaction network metrics, both in the NBD and SBD of both proteins, as identified in this paper, could provide plausible explanations of ligand specific signal propagation patterns, as well as ligand induced regulatory mechanisms. We expect that this study can offer useful information for the design of next generation Hsp70 allosteric modulators.

## Figures and Tables

**Table 1:** Total binding free energy results. Tabulated summary of energy term contributions and the resultant total binding free energy.

Protein		Energy Terms				ΔG_binding_ (kJ·mol^−1^)
		Δ*E*_vdW_	Δ*E*_elec_	ΔG_polar_	ΔG_nonpolar_	
Hsp72-SANC00132 complex	Run1	−182.161 ± 0.275	−75.159 ± 0.289	167.323 ± 0.497	−15.361 ± 0.018	−105.338 ± 0.378
	Run2	−201.464 ± 0.348	−101.914 ± 0.243	194.286 ± 0.231	−17.317 ± 0.015	−126.402 ± 0.328
Hsp72 endo-complex	Run1	−189.941 ± 0.311	−67.232 ± 0.257	195.474 ± 0.263	−16.650 ± 0.017	−78.365 ± 0.321
	Run2	−201.786 ± 0.365	−131.232 ± 0.260	241.696 ± 0.303	−16.901 ± 0.016	−108.205 ± 0.330
Hsc70-SANC00132 complex	Run1	−155.398 ± 0.331	−64.820 ± 1.132	161.916 ± 1.473	−15.631 ± 0.028	−73.973 ± 0.547
	Run2	−137.056 ± 0.277	−77.472 ± 0.305	155.394 ± 0.390	−15.298 ± 0.017	−74.006 ± 0.542
Hsc70 endo-complex	Run1	−196.548 ± 0.271	−49.657 ± 0.290	143.562 ± 0.318	−17.164 ± 0.017	−119.818 ± 0.282
	Run2	−189.970 ± 0.248	−43.352 ± 0.204	140.112 ± 0.252	−16.014 ± 0.016	−109.226 ± 0.255

**Table 2:**
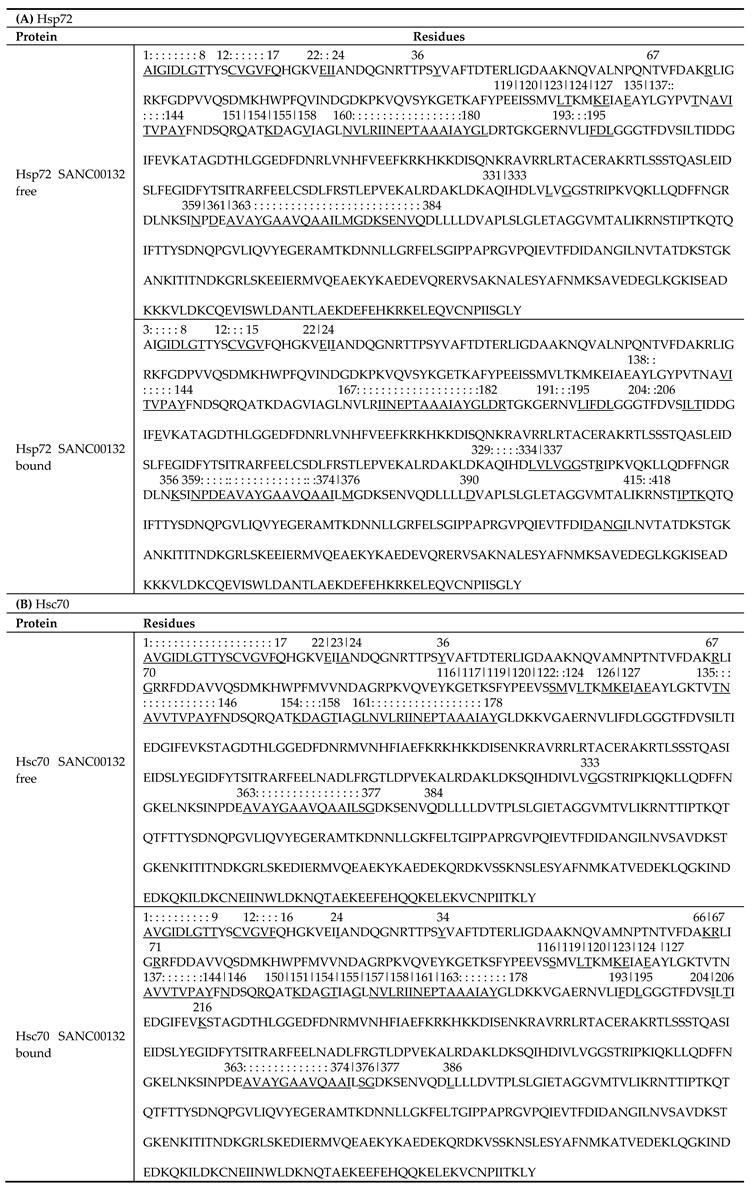
(**A** and **B**) Tabulated summary of amino-acids that yielded low average L values averaged across all ligand-free and ligand-bound simulation systems of each protein. Identified residues and respective positions were mapped within the primary sequence. Residues are underlined while positions are indicated above the residues. Continuous stretches of residue positions are indicated by colon punctuations. Residue numbering is according to *E. coli* DnaK sequence (UniProtKB ID: P0A6Y8). Equivalent residue numbering from complete Hsp72 and Hsc70 sequences are tabulated in Table S8A,B.

**Table 3:**
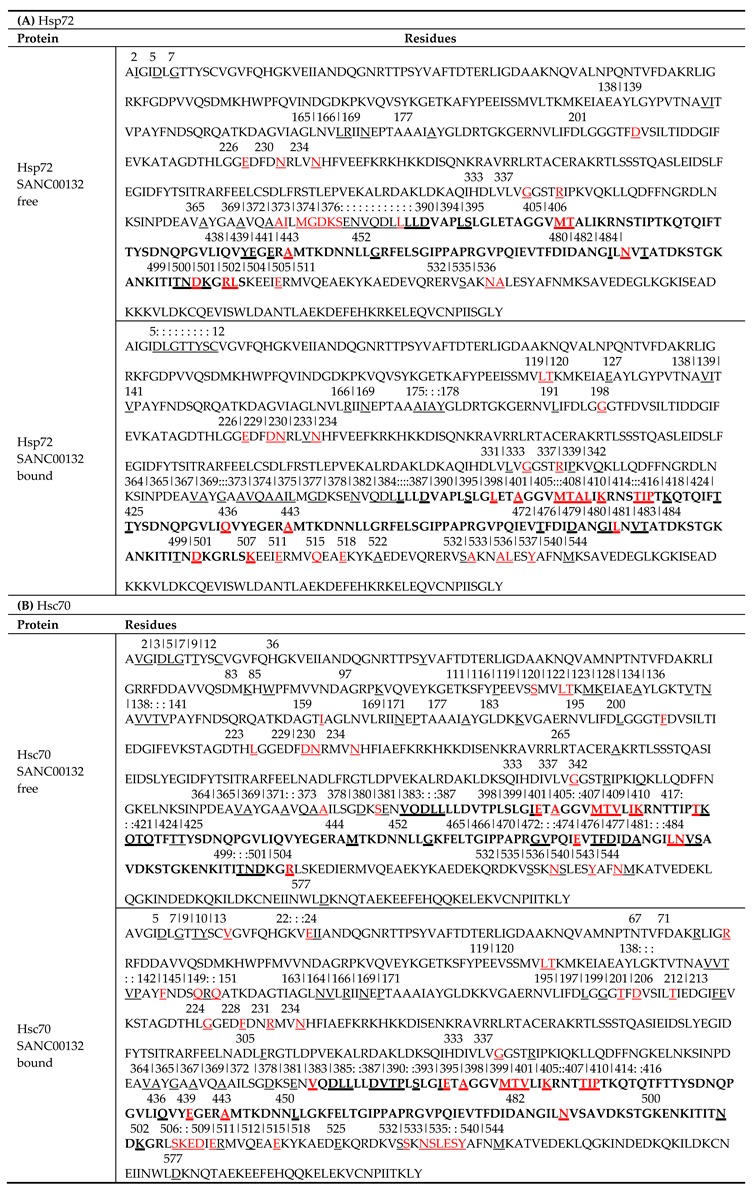
(**A** and **B**) Tabulated summary of residues possessing high average BC indices across all ligand-free and ligand-bound trajectories of each protein. Identified residues and respective positions were mapped within the primary sequence. Residues are underlined while positions and numberings are indicated above the residues. Numbering is based on *E. coli* DnaK template sequence (UniProtKB ID: P0A6Y8). Continuous stretches of residue positions are indicated by colon punctuations. Residues highlighted in red represent residues agreeing with PRS results on *E. coli* DnaK [18]. Residues existing within the ligand binding site are shown in bold. Corresponding residue numbering from complete sequences of respective proteins are shown in Table S9A,B.

**Table 4:** Pairwise comparison between BC, L^−1^ and RMSF^−1^ (100 ns and last 15 ns time frames) using Pearson’s correlation coefficient, apo run1, (**ii**).

Protein	L vs. RMSF (100 ns)	L vs. RMSF (last 15 ns)	L^−1^ vs. BC	RMSF^−1^ (100 ns) vs. BC	RMSF^−1^ (last 15 ns) vs. BC
Hsp72 apo Run1	0.53	0.45	0.39	0.12	0.10
Hsp72 apo Run2	0.57	0.53	0.39	0.07	0.11
Hsp72-SANC00132 Run1	0.43	0.77	0.51	0.15	0.37
Hsp72-SANC00132 Run2	0.64	0.74	0.49	0.12	0.27
Hsp72 endo-apo Run1	0.56	0.80	0.45	0.19	0.30
Hsp72 endo-apoRun2	0.61	0.78	0.45	0.24	0.28
Hsp72 endo-complex Run1	0.69	0.38	0.43	0.23	0.03
Hsp72 endo-complex Run2	0.58	0.68	0.48	0.16	0.28
Hsc70 apo Run1	0.58	0.87	0.49	0.10	0.37
Hsc70 apo Run2	0.62	0.72	0.48	0.15	0.13
Hsc70-SANC00132 Run1	0.74	0.77	0.40	0.32	0.30
Hsc70-SANC00132 Run2	0.70	0.72	0.40	0.09	0.32
Hsc70 endo-apo Run1	0.59	0.72	0.46	0.12	0.30
Hsc70 endo-apo Run2	0.76	0.80	0.42	0.25	0.28
Hsc70 endo-complex Run1	0.60	0.77	0.43	0.15	0.22
Hsc70 endo-complex Run2	0.51	0.58	0.45	0.04	0.30

## Supplementary Materials

The supplementary materials are available online.

## References

[1] Zugazagoitia J., Guedes C., Ponce S., Ferrer I., Molina-Pinelo S., Paz-Ares L. (2016). Current Challenges in Cancer Treatment. Clin. Ther..

[2] Grivennikov S.I., Greten F.R., Karin M. (2010). Immunity, Inflammation, and Cancer. Cell.

[3] Granato M., Santarelli R., Gonnella R., Farina A., Trivedi P., Faggioni A., Cirone M. (2015). Targeting of prosurvival pathways as therapeutic approaches against primary effusion lymphomas: Past, present, and Future. Biomed. Res. Int..

[4] Murphy M.E. (2013). The HSP70 family and cancer. Carcinogenesis.

[5] Hartl F.U. (1996). Molecular chaperones in cellular protein folding. Nature.

[6] Daugaard M., Rohde M., Jäättelä M. (2007). The heat shock protein 70 family: Highly homologous proteins with overlapping and distinct functions. FEBS Lett..

[7] Tannock I.F., Rotin D. (1989). Acid pH in tumors and its potential for therapeutic exploitation. Cancer Res..

[8] Navrátilová J., Hankeová T., Beneš P., Šmarda J. (2013). Low-Glucose Conditions of Tumor Microenvironment Enhance Cytotoxicity of Tetrathiomolybdate to Neuroblastoma Cells. Nutr. Cancer.

[9] Eales K.L., Hollinshead K.E.R., Tennant D.A. (2016). Hypoxia and metabolic adaptation of cancer cells. Oncogenesis.

[10] Garrido C., Brunet M., Didelot C., Zermati Y., Schmitt E., Kroemer G. (2006). Heat Shock Proteins 27 and 70: Anti-Apoptotic Proteins with Tumorigenic Properties. Cell Cycle.

[11] Gabai V.L., Yaglom J.A., Waldman T., Sherman M.Y. (2009). Heat shock protein Hsp72 controls oncogene-induced senescence pathways in cancer cells. Mol. Cell. Biol..

[12] Ciocca D.R., Calderwood S.K. (2005). Heat shock proteins in cancer: Diagnostic, prognostic, predictive, and treatment implications. Cell Stress Chaperones.

[13] Pratt W.B., Toft D.O. (2003). Regulation of signaling protein function and trafficking by the hsp90/hsp70-based chaperone machinery. Exp. Biol. Med..

[14] Wegele H., Müller L., Buchner J. (2004). Hsp70 and Hsp90—A relay team for protein folding. Reviews of Physiology, Biochemistry and Pharmacology.

[15] Garrido C., Ottavi P., Fromentin A., Hammann A., Arrigo A.P.A., Chauffert B., Mehlen P. (1997). HSP27 as a mediator of confluence-dependent resistance to cell death induced by anticancer drugs. Cancer Res..

[16] Powers M.V., Clarke P.A., Workman P. (2008). Dual Targeting of HSC70 and HSP72 Inhibits HSP90 Function and Induces Tumor-Specific Apoptosis. Cancer Cell.

[17] Zhuravleva A., Clerico E.M., Gierasch L.M. (2012). An Interdomain Energetic Tug-of-War Creates the Allosterically Active State in Hsp70 Molecular Chaperones. Cell.

[18] Penkler D., Sensoy Ö., Atilgan C., Tastan Bishop Ö. (2017). Perturbation-Response Scanning Reveals Key Residues for Allosteric Control in Hsp70. J. Chem. Inf. Model..

[19] Sharma D., Masison D.C. (2009). Hsp70 structure, function, regulation and influence on yeast prions. Protein Pept. Lett..

[20] Zhu X., Zhao X., Burkholder W.F., Gragerov A., Ogata C.M., Gottesman M.E., Hendrickson W.A. (1996). Structural analysis of substrate binding by the molecular chaperone DnaK. Science.

[21] Bukau B., Horwich A.L. (1998). The Hsp70 and Hsp60 Chaperone Machines. Cell.

[22] Zhuravleva A., Gierasch L.M. (2015). Substrate-binding domain conformational dynamics mediate Hsp70 allostery. Proc. Natl. Acad. Sci..

[23] Slepenkov S.V., Witt S.N. (1998). Kinetics of the Reactions of the *Escherichia coli* Molecular Chaperone DnaK with ATP: Evidence That a Three-Step Reaction Precedes ATP Hydrolysis. Biochemistry.

[24] Hatherley R., Brown D.K., Glenister M., Tastan Bishop Ö. (2016). PRIMO: An Interactive Homology Modeling Pipeline. PLoS ONE.

[25] Ferraro M., D’Annessa I., Moroni E., Morra G., Paladino A., Rinaldi S., Compostella F., Colombo G. (2018). Allosteric Modulators of HSP90 and HSP70: Dynamics Meets Function through Structure-Based Drug Design. J. Med. Chem..

[26] Karzai A.W., McMacken R. (1996). A bipartite signaling mechanism involved in DnaJ-mediated activation of the Escherichia coli DnaK protein. J. Biol. Chem..

[27] English C.A., Sherman W., Meng W., Gierasch L.M. (2017). The Hsp70 interdomain linker is a dynamic switch that enables allosteric communication between two structured domains. J. Biol. Chem..

[28] Zhuravleva A., Gierasch L.M. (2011). Allosteric signal transmission in the nucleotide-binding domain of 70-kDa heat shock protein (Hsp70) molecular chaperones. Proc. Natl. Acad. Sci. USA..

[29] Hayer-Hartl M., Martin J., Hartl F., Christen P., Kuriyan J. (1995). Asymmetrical interaction of GroEL and GroES in the ATPase cycle of assisted protein folding. Science.

[30] Harrison C. (2003). GrpE, a nucleotide exchange factor for DnaK. Cell Stress Chaperones.

[31] Liu Y., Gierasch L.M., Bahar I. (2010). Role of Hsp70 ATPase Domain Intrinsic Dynamics and Sequence Evolution in Enabling its Functional Interactions with NEFs. PLoS Comput. Biol..

[32] Woo H.J., Jiang J., Lafer E.M., Sousa R. (2009). ATP-induced conformational changes in Hsp70: Molecular dynamics and experimental validation of an in silico predicted conformation. Biochemistry.

[33] Swain J.F., Schulz E.G., Gierasch L.M. (2006). Direct comparison of a stable isolated Hsp70 substrate-binding domain in the empty and substrate-bound states. J. Biol. Chem..

[34] Gassler C.S., Wiederkehr T., Brehmer D., Bukau B., Mayer M.P. (2001). Bag-1M accelerates nucleotide release for human Hsc70 and Hsp70 and can act concentration-dependent as positive and negative cofactor. J. Biol. Chem..

[35] Kityk R., Kopp J., Sinning I., Mayer M.P. (2012). Structure and Dynamics of the ATP-Bound Open Conformation of Hsp70 Chaperones. Mol. Cell.

[36] Leu J.I.J., Pimkina J., Frank A., Murphy M.E., George D.L. (2009). A Small Molecule Inhibitor of Inducible Heat Shock Protein 70. Mol. Cell.

[37] Brodsky J.L. (1999). Selectivity of the molecular chaperone-specific immunosuppressive agent 15-deoxyspergualin: Modulation of HSC70 ATPase activity without compromising DnaJ chaperone interactions. Biochem. Pharmacol..

[38] Dal Piaz F., Cotugno R., Lepore L., Vassallo A., Malafronte N., Lauro G., Bifulco G., Belisario M.A., De Tommasi N. (2013). Chemical proteomics reveals HSP70 1A as a target for the anticancer diterpene oridonin in Jurkat cells. J. Proteomics.

[39] Hassan A.Q., Kirby C.A., Zhou W., Schuhmann T., Kityk R., Kipp D.R., Baird J., Chen J., Chen Y., Chung F. (2015). The Novolactone Natural Product Disrupts the Allosteric Regulation of Hsp70. Chem. Biol..

[40] Pettinger J., Le Bihan Y.V., Widya M., van Montfort R.L.M., Jones K., Cheeseman M.D. (2017). An Irreversible Inhibitor of HSP72 that Unexpectedly Targets Lysine-56. Angew. Chem. Int. Ed. Engl..

[41] Jones A.M., Westwood I.M., Osborne J.D., Matthews T.P., Cheeseman M.D., Rowlands M.G., Jeganathan F., Burke R., Lee D., Kadi N. (2016). A fragment-based approach applied to a highly flexible target: Insights and challenges towards the inhibition of HSP70 isoforms. Sci. Rep..

[42] Rodina A., Patel P.D., Kang Y., Patel Y., Baaklini I., Wong M.J.H., Taldone T., Yan P., Yang C., Maharaj R. (2013). Identification of an allosteric pocket on human hsp70 reveals a mode of inhibition of this therapeutically important protein. Chem. Biol..

[43] Halgren T.A. (2009). Identifying and Characterizing Binding Sites and Assessing Druggability. J. Chem. Inf. Model..

[44] Leu J.I.J., Zhang P., Murphy M.E., Marmorstein R., George D.L. (2014). Structural basis for the inhibition of HSP70 and DnaK chaperones by small-molecule targeting of a C-terminal allosteric pocket. ACS Chem. Biol..

[45] 45 Stetz G., Verkhivker G.M. (2016). Probing Allosteric Inhibition Mechanisms of the Hsp70 Chaperone Proteins Using Molecular Dynamics Simulations and Analysis of the Residue Interaction Networks. J. Chem. Inf. Model..

[46] Penkler D., Tastan Bishop Ö. (2019). Modulation of Human Hsp90α Conformational Dynamics by Allosteric Ligand Interaction at the C-Terminal Domain. Sci. Rep..

[47] Hatherley R., Brown D.K., Musyoka T.M., Penkler D.L., Faya N., Lobb K.A., Tastan Bishop Ö. (2015). SANCDB: A South African natural compound database. J. Cheminform..

[48] Hu J.F., Fan H., Xiong J., Wu S.B. (2011). Discorhabdins and Pyrroloiminoquinone-Related Alkaloids. Chem. Rev..

[49] Antunes E.M., Beukes D.R., Kelly M., Samaai T., Barrows L.R., Marshall K.M., Sincich C., Davies-Coleman M.T. (2004). Cytotoxic Pyrroloiminoquinones from Four New Species of South African Latrunculid Sponges. J. Nat. Prod..

[50] Harris E., Strope J., Beedie S., Huang P., Goey A., Cook K., Schofield C., Chau C., Cadelis M., Copp B. (2018). Preclinical Evaluation of Discorhabdins in Antiangiogenic and Antitumor Models. Mar. Drugs.

[51] Söding J., Biegert A., Lupas A.N. (2005). The HHpred interactive server for protein homology detection and structure prediction. Nucleic Acids Res..

[52] Altschul S.F., Madden T.L., Schäffer A.A., Zhang J., Zhang Z., Miller W., Lipman D.J. (1997). Gapped BLAST and PSI-BLAST: A new generation of protein database search programs. Nucleic Acids Res..

[53] Shen M.Y., Sali A. (2006). Statistical potential for assessment and prediction of protein structures. Protein Sci..

[54] Ramachandran G.N., Ramakrishnan C., Sasisekharan V. (1963). Stereochemistry of polypeptide chain configurations. J. Mol. Biol..

[55] Eisenberg D., Lüthy R., Bowie J.U. (1997). VERIFY3D: Assessment of protein models with three-dimensional profiles. Methods Enzymol..

[56] Wiederstein M., Sippl M.J. (2007). ProSA-web: Interactive web service for the recognition of errors in three-dimensional structures of proteins. Nucleic Acids Res..

[57] Trott O., Olson A.J. (2010). AutoDock Vina: Improving the speed and accuracy of docking with a new scoring function, efficient optimization, and multithreading. J. Comput. Chem..

[58] Wang R., Lai L., Wang S. (2002). Further development and validation of empirical scoring functions for structure-based binding affinity prediction. J. Comput. Aided. Mol. Des..

[59] Yang J.M., Chen Y.F., Shen T.W., Kristal B.S., Hsu D.F. (2005). Consensus Scoring Criteria for Improving Enrichment in Virtual Screening. J. Chem. Inf. Model..

[60] Lipinski C.A. (2004). Lead- and drug-like compounds: The rule-of-five revolution. Drug Discov. Today Technol..

[61] Baell J.B. (2015). Screening-Based Translation of Public Research Encounters Painful Problems. ACS Med. Chem. Lett..

[62] Capuzzi S.J., Muratov E.N., Tropsha A. (2017). Phantom PAINS: Problems with the Utility of Alerts for pan-assay interference Compounds. J. Chem. Inf. Model..

[63] Shrestha J.P., Subedi Y.P., Chen L., Chang C.W.T. (2015). A mode of action study of cationic anthraquinone analogs: A new class of highly potent anticancer agents. Medchemcomm.

[64] Swain J.F., Dinler G., Sivendran R., Montgomery D.L., Stotz M., Gierasch L.M. (2007). Hsp70 chaperone ligands control domain association via an allosteric mechanism mediated by the interdomain linker. Mol. Cell.

[65] Ma B., Kumar S., Tsai C.J., Nussinov R. (1999). Folding funnels and binding mechanisms. Protein Eng. Des. Sel..

[66] Tsai C.J., Nussinov R. (2014). The free energy landscape in translational science: How can somatic mutations result in constitutive oncogenic activation?. Phys. Chem. Chem. Phys..

[67] Kollman P.A., Massova I., Reyes C., Kuhn B., Huo S., Chong L., Lee M., Lee T., Duan Y., Wang W. (2000). Calculating structures and free energies of complex molecules: Combining molecular mechanics and continuum models. Acc. Chem. Res..

[68] Kumari R., Kumar R., Lynn A. (2014). G-mmpbsa-A GROMACS tool for high-throughput MM-PBSA calculations. J. Chem. Inf. Model..

[69] Abraham M.J., Murtola T., Schulz R., Páll S., Smith J.C., Hess B., Lindahl E. (2015). GROMACS: High performance molecular simulations through multi-level parallelism from laptops to supercomputers. SoftwareX.

[70] BIOVA Discovery Studio. http://accelrys.com/products/collaborative-science/biovia-discovery-studio/.

[71] Laskowski R.A., Swindells M.B. (2011). LigPlot+: Multiple ligand-protein interaction diagrams for drug discovery. J. Chem. Inf. Model..

[72] Li H.Y., Wang J.H. (2009). Folding rate prediction using complex network analysis for proteins with two- and three-state folding kinetics. J. Biomed. Sci. Eng..

[73] del Sol A., Fujihashi H., Amoros D., Nussinov R. (2006). Residues crucial for maintaining short paths in network communication mediate signaling in proteins. Mol. Syst. Biol..

[74] Santoni D., Paci P., Paola L.D., Giuliani A. (2016). Are Proteins Just Coiled Cords? Local and Global Analysis of Contact Maps Reveals the Backbone-Dependent Nature of Proteins. Ingenta Connect.

[75] Penkler D., Atilgan C., Tastan Bishop Ö. (2018). Allosteric Modulation of Human Hsp90α Conformational Dynamics. J. Chem. Inf. Model..

[76] Qi R., Sarbeng E.B., Liu Q., Le K.Q., Xu X., Xu H., Yang J., Wong J.L., Vorvis C., Hendrickson W.A. (2013). Allosteric opening of the polypeptide-binding site when an Hsp70 binds ATP. Nat. Struct. Mol. Biol..

[77] Zhang P., Leu J.I.-J., Murphy M.E., George D.L., Marmorstein R. (2014). Crystal Structure of the Stress-Inducible Human Heat Shock Protein 70 Substrate-Binding Domain in Complex with Peptide Substrate. PLoS ONE.

[78] Sali A., Blundell T.L. (1993). Comparative protein modelling by satisfaction of spatial restraints. J. Mol. Biol..

[79] Laskowski R.A., MacArthur M.W., Moss D.S., Thornton J.M. (1993). IUCr PROCHECK: A program to check the stereochemical quality of protein structures. J. Appl. Crystallogr..

[80] Morris G.M., Huey R., Lindstrom W., Sanner M.F., Belew R.K., Goodsell D.S., Olson A.J. (2009). AutoDock4 and AutoDockTools4: Automated docking with selective receptor flexibility. J. Comput. Chem..

[81] Sterling T., Irwin J.J. (2015). ZINC 15--Ligand Discovery for Everyone. J. Chem. Inf. Model..

[82] Duan Y., Wu C., Chowdhury S., Lee M.C., Xiong G., Zhang W., Yang R., Cieplak P., Luo R., Lee T. (2003). A point-charge force field for molecular mechanics simulations of proteins based on condensed-phase quantum mechanical calculations. J. Comput. Chem..

[83] Sousa da Silva A.W., Vranken W.F. (2012). ACPYPE—AnteChamber PYthon Parser interface. BMC Res. Notes.

[84] Humphrey W., Dalke A., Schulten K. (1996). VMD: Visual molecular dynamics. J. Mol. Graph..

[85] Musyoka T.M., Kanzi A.M., Lobb K.A., Tastan Bishop Ö. (2016). Structure Based Docking and Molecular Dynamic Studies of Plasmodial Cysteine Proteases against a South African Natural Compound and its Analogs. Sci. Rep..

[86] Musyoka T.M., Kanzi A.M., Lobb K.A., Tastan Bishop Ö. (2016). Analysis of non-peptidic compounds as potential malarial inhibitors against *Plasmodial* cysteine proteases via integrated virtual screening workflow. J. Biomol. Struct. Dyn..

[87] Brown D., Penkler D., Sheik Amamuddy O., Ross C., Atilgan A.R., Atilgan C., Tastan Bishop Ö. (2017). MD-TASK: A software suite for analyzing molecular dynamics trajectories. Bioinformatics.

