# Supplementary material for: Discorhabdin N, a South African Natural Compound, for Hsp72 and Hsc70 Allosteric Modulation: Combined Study of Molecular Modeling and Dynamic Residue Network Analysis
Source: Molecules. 2019 Jan 6;24(1):188. doi: 10.3390/molecules24010188 (PMC6337312; doi:10.3390/molecules24010188)
Supplement: Supplementary file 1 [file molecules-24-00188-s001.zip › SD/Amusengeri_TastanBishop_Supplementary_December2018.pdf]

Supplementary Material

Discorhabdin N, a South African natural compound, for Hsp72 and Hsc70 allosteric modulation: Combined study of molecular modeling and dynamic residue network analysis

Arnold Amusengeri[1a] and Özlem Tastan Bishop[1b]*

[1]Research Unit in Bioinformatics (RUBi), Department of Biochemistry and Microbiology, Rhodes University, Grahamstown, 6140, South Africa

*** Correspondence:**
Özlem Tastan Bishop
email: O.TastanBishop@ru.ac.za

Table S1: Structure validation results: Tabulated summary of Z-dope, Ramachandran, ProSA and Verify-3D results.

Protein	Z-Dope score	PROCHECK (Ramachandran Plot)				ProSA Z-Score	Verify 3D	
		Number of residues in favoured region (Percentage value)	Number of residues in additional allowed region (Percentage value)	Number of residues in generously allowed region (Percentage value)	Number of residues in disallowed region (Percentage value)		Score	Comment
Hsp72	-0.51	93.70%	5.20%	0.70%	0.40%	-10.98	85.81%	Pass
Hsc70	-0.56	94.10%	4.80%	0.50%	0.50%	-11.07	83.99%	Pass

Hsp72

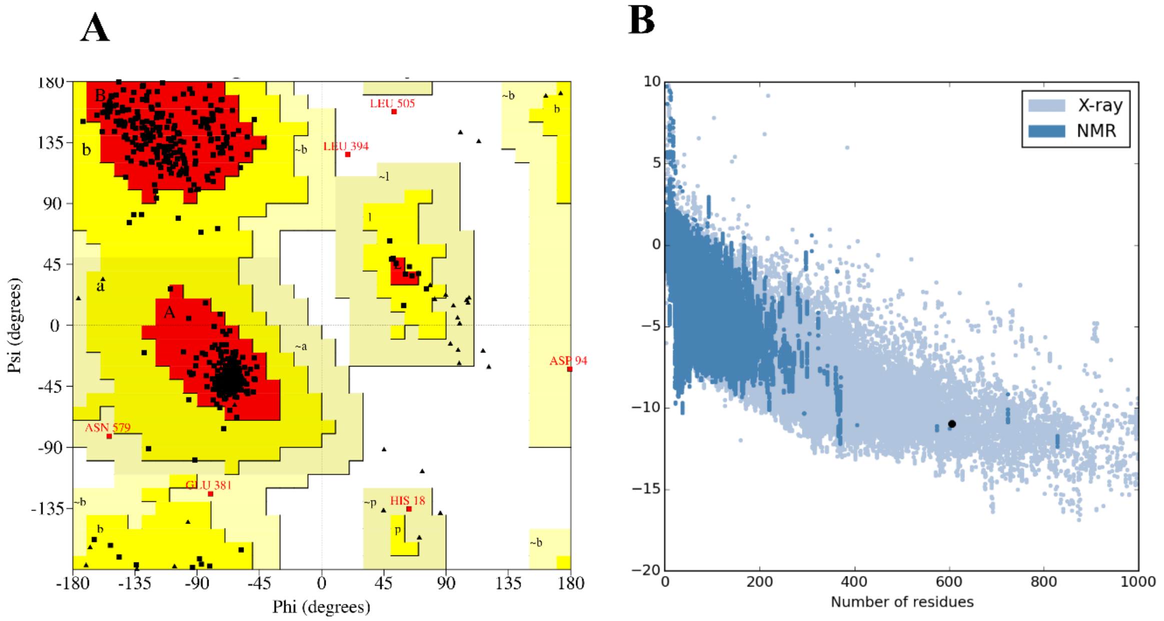

Hsc70

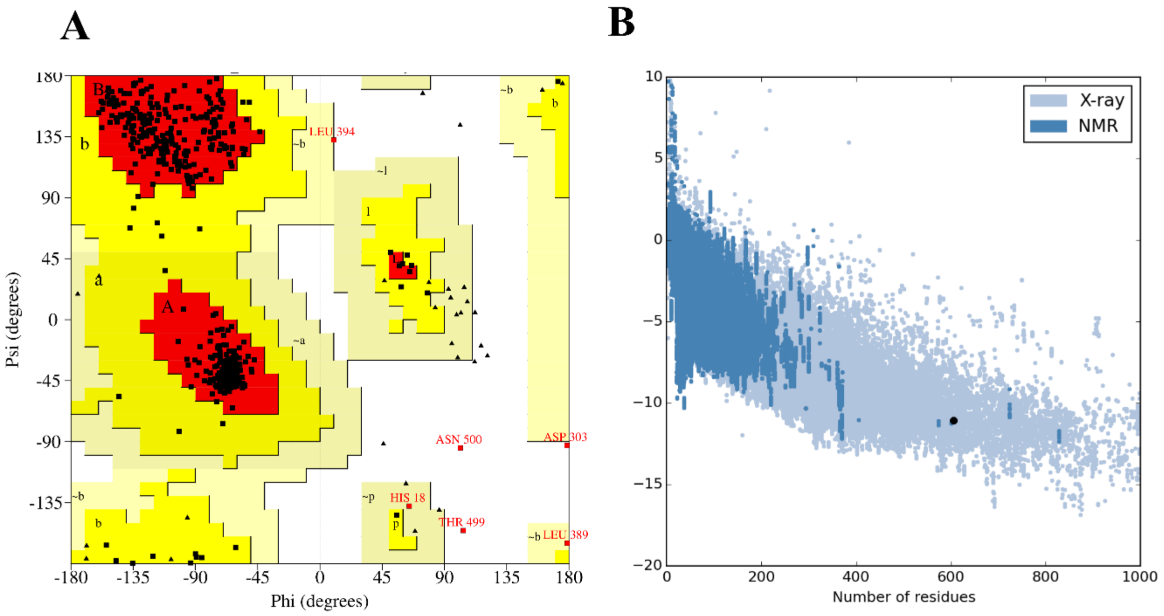

Figure S1: Model validation summary. (A) Ramachandran plots. In both proteins, more than 90% of all residues occupied the most favoured region (Hsp72: 93.70%, Hsc70: 94.10%). **(B) ProSA results.** Both proteins recorded low Z-Score values (Hsp72: -10.98, Hsc70: -11.07) that were within the range of high resolution crystallized proteins.

Hsp72

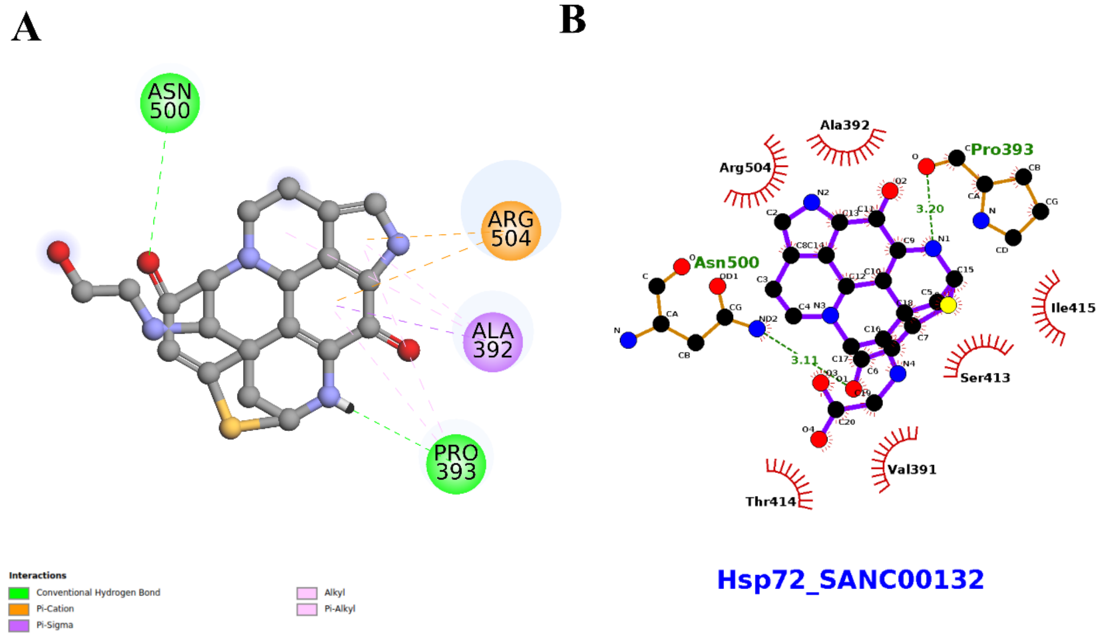

Hsc70

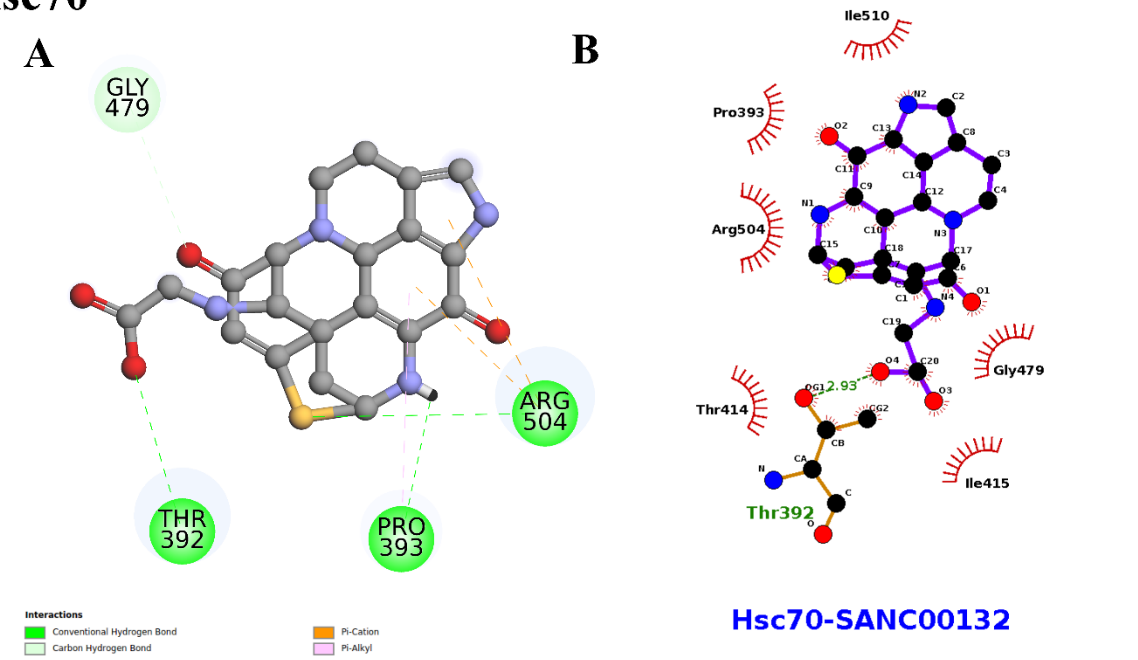

Figure S2: Molecular docking interactions visualised using (A) Discovery studio visualizer, and (B) LigPlot+. (A) SANC00132 is displayed in ball and stick while non-bonded interactions are shown as dashed lines. (B) SANC00132 is represented in ball and stick while bonds are shown sticks (purple solid lines). Hydrogen bonds are shown as dashed green lines while protein residues making hydrogen bond connections are represented in ball and stick.

Table S2: Tabulated results from Vina and XScore scoring tools.

Compound	Molecular weight (g/mol)	Protein	Vina		X-Score				
			Vina Score (Kcal/mol)	Rank	HPScore (pKd)	HMScore (pKd)	HSScore (pKd)	Average (pKd)	Rank
SANC00132	396.30	Hsp72	-6.9	7	5.56	5.48	5.48	5.51	14
		Hsc70	-7.1	11	5.78	5.91	5.53	5.74	5

Table S3: Post-docking analysis results. Tabulated results of (A) Hsp72 and (B) Hsc70 residues interacting with SANC00132.

(A)

Hsp72	
Hsp72 interacting residues (DNAK numbering)	Hsp72 complete sequence equivalent
ALA392	ALA397
PRO393	PRO398
ASN500	ASN505
ARG504	ARG509

(B)

Hsc70	
Hsc70 interacting residues (DNAK numbering)	Hsc70 complete sequence equivalent
THR392	THR397
PRO393	PRO398
GLY479	GLY484
ARG504	ARG509

Table S4: Results of computed molecular descriptors using FAF-Drugs4 web-server. SANC00132 passed the Lipinski tests for drug likeness (RO5) including Molecular mass < 500 Dalton: MW = 410.45, Lipophilicity (LogP) < 5: logP = -2.36, Hydrogen bond donors < 5: HBD = 4, and Hydrogen bond acceptors < 10: HBA = 8.

**Ligand**	**SANC00132**
**MW**	410.45
**logP**	-2.36
**logD**	-3.76
**logSw**	-0.78
**tPSA**	147.24
**RotatableB**	3
**RigidB**	31
**Flexibility**	0.09
**HBD**	4
**HBA**	8
**HBD_HBA**	12
**Rings**	1
**MaxSizeRing**	24
**NumCharges**	2
**TotalCharge**	0
**HeavyAtoms**	29
**CarbonAtoms**	20
**HeteroAtoms**	9
**ratioH/C**	0.45
**Lipinski_Violation**	0
**Solubility(mg/l)**	188397.59
**SolubilityForecastIndex**	Good Solubility
**Oral_Bioavailability_VEBER**	Good
**Oral_Bioavailability_EGAN**	Good
**TrafficLights**	3
**4_400**	good
**3_75**	good
**Phospholipidosis**	NonInducer
**Fsp3**	0.45
**StereoCenters**	5
**PAINS_Filter_A**	0
**PAINS_Filter_B**	0
**PAINS_Filter_C**	0
**Result**	Accepted

Table S5: Tabulated RMSD convergence values

Protein		RMSD convergence value
Hsp72 apo run1	Run1	~1.5 nm
	Run2	~1.2 nm
Hsp72 endo-apo	Run1	~1.2 nm
	Run2	~1.9 nm
Hsc70 apo	Run1	~1.5 nm
	Run2	~1.7 nm
Hsc70 endo-apo	Run1	~1.6 nm
	Run2	~1.3 nm
Hsp72-SANC00132 complex	Run1	~1.5 nm
	Run2	~1.5 nm
Hsp72 endo-complex	Run1	~0.7 nm
	Run2	~1.5 nm
Hsc70-SANC00132 complex	Run1	~1.2 nm
	Run2	~1.2 nm
Hsc70 endo-complex	Run1	~1.5 nm
	Run2	~1.5 nm

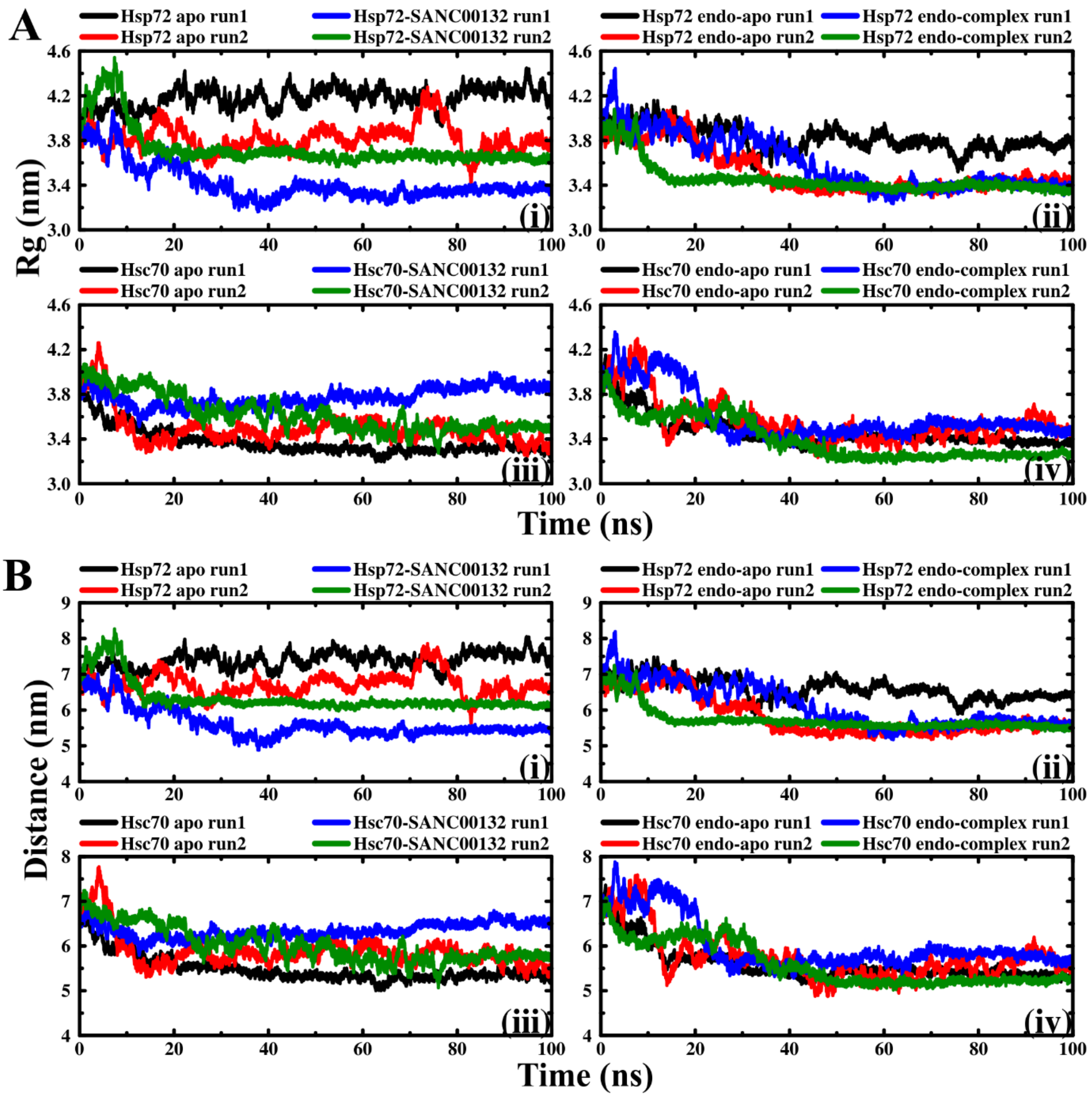

Figure S3: (A) Time dependent radius of gyration (Rg) of Hsp72 and Hsc70 during 100ns simulation. (i) Hsp72 Set1, (ii) Hsp72 Set2, (iii) Hsc70 Set1, (iv) Hsc70 Set2. **(B) Time-dependent distance between the centre of mass of the NBD and that of the SBD.** (i) Hsp72 Set1, (ii) Hsp72 Set2, (iii) Hsc70 Set1, (iv) Hsc70 Set2. Colour code: Black and red: inhibitor-free, Blue and green: inhibitor-bound.

Table S6: Tabulated values of the percentage variance contributed by the first two principal components. Also displayed are respective trace values of diagonalized covariance matrices of each MD trajectory.

Protein systems		Percentage variance accounted by each component		Trace value / cumulative sum of eigenvalues (nm^2)	Trace value variance between Run1 and Run2
		PC1 (%)	PC2 (%)		
Hsp72 apo	Run1	54.10	24.96	520.860	3854.51
	Run2	55.12	27.31	433.059	
Hsp72-SANC00132 complex	Run1	57.54	24.03	263.135	353.89
	Run2	63.10	16.64	236.531	
Hsp72 endo-apo	Run1	46.01	23.76	247.669	15335.08
	Run2	70.88	19.48	422.798	
Hsp72 endo-complex	Run1	70.06	18.35	635.812	1522120.50
	Run2	62.00	13.95	84.2316	
Hsc70 apo	Run1	71.04	8.80	99.7293	5550.44
	Run2	48.94	29.23	205.09	
Hsc70-SANC00132 complex	Run1	47.79	21.59	129.313	4895.25
	Run2	58.47	26.04	228.26	
Hsc70 endo-apo	Run1	59.96	27.82	209.165	65.38
	Run2	42.19	22.91	197.73	
Hsc70 endo-complex	Run1	78.62	9.45	350.493	37.27
	Run2	75.85	13.66	341.859	
					Total variance =182212.3<br><br>Average standard deviation (√ (Total variance)) = 426.86

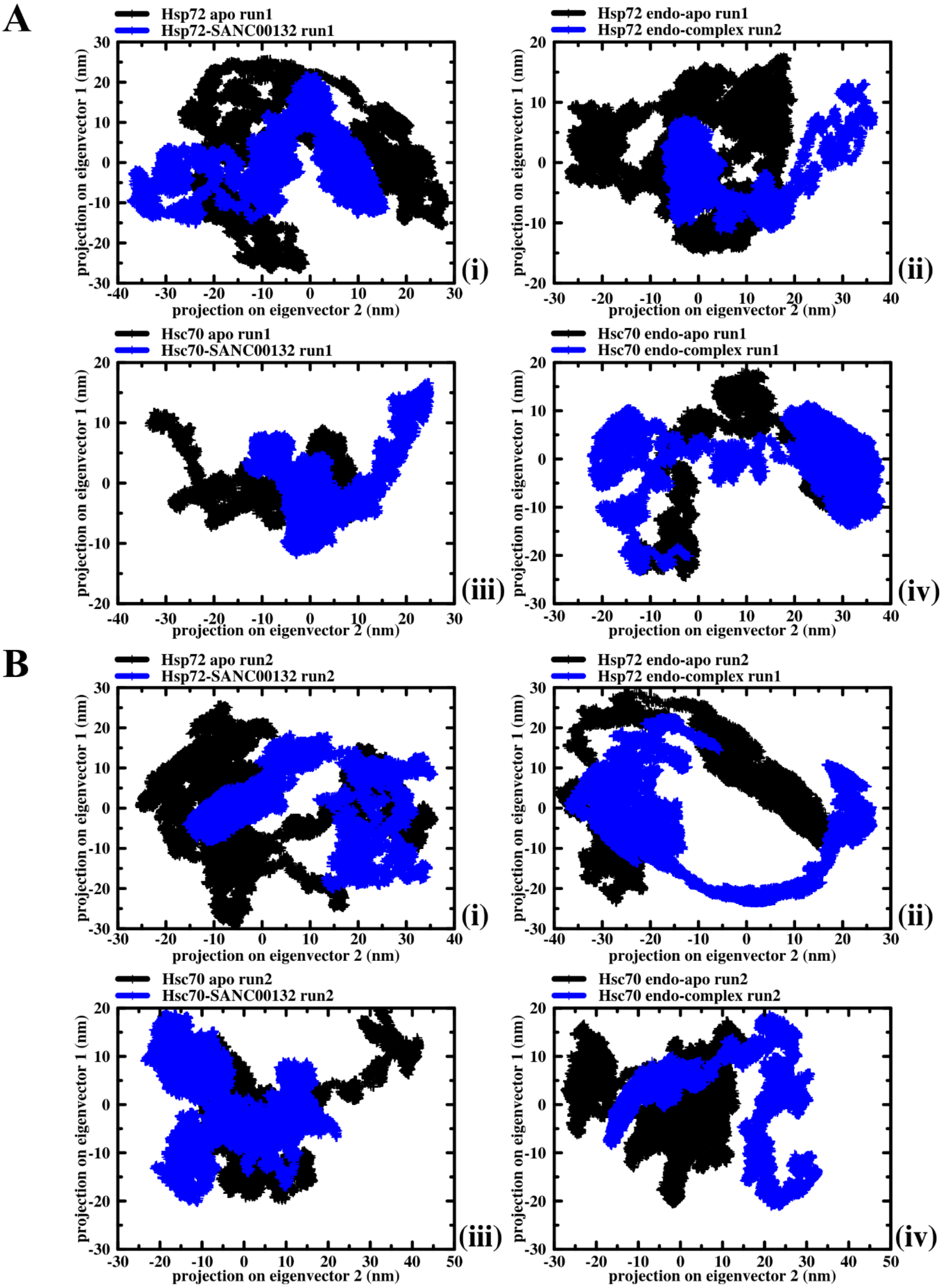

Figure S4: 2D projection of first and second principal components. Colour key: Black: inhibitor-free, Blue; inhibitor-bound. **A:** Initial simulations, **B:** Duplicate simulations.

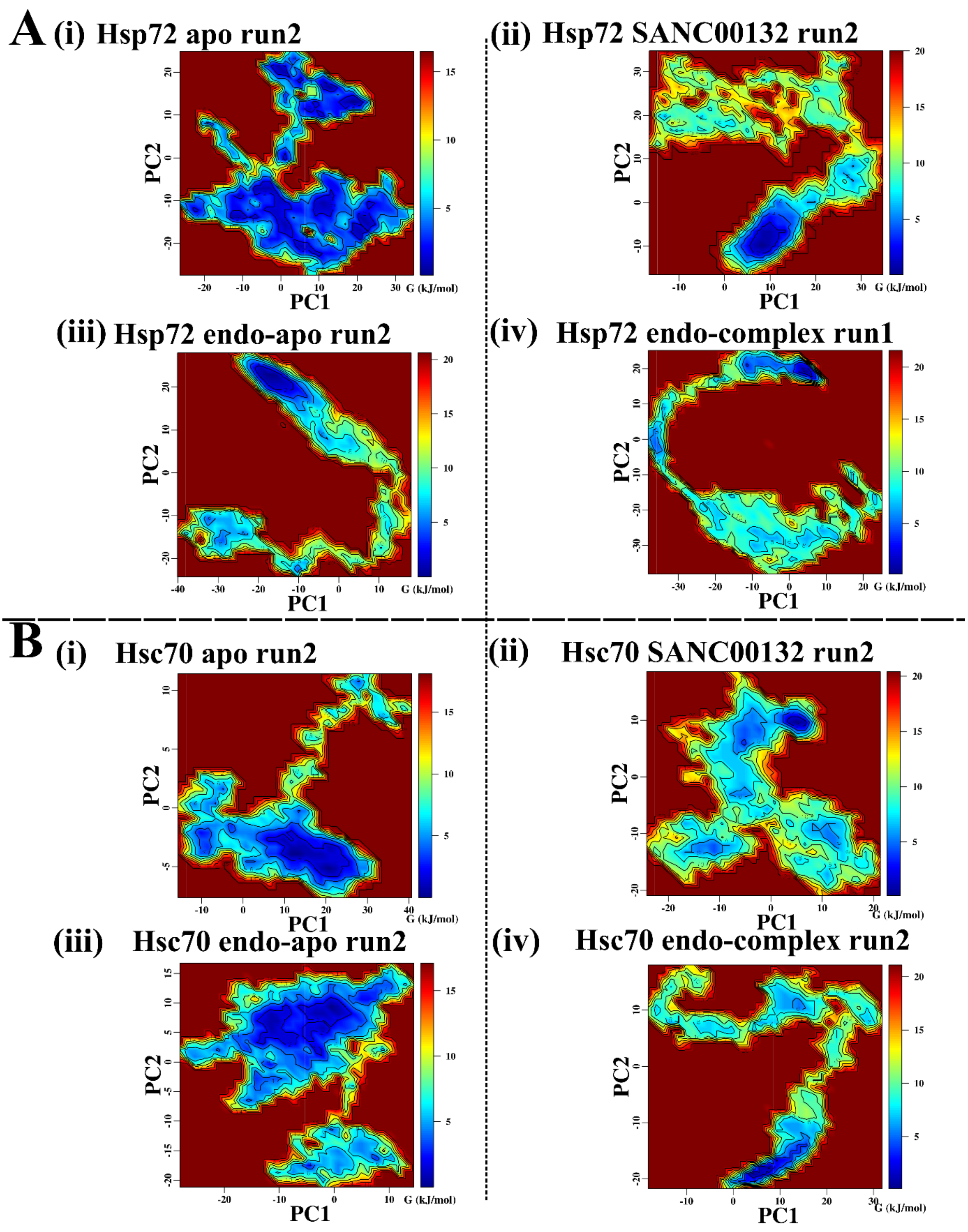

Figure S5: Free energy landscape projections along the first and second principal components. A: Hsp72: (i) Hsp72 apo Run2, (ii) Hsp72-SANC00132 Run2, (iii) Hsp72 endo-apo Run2 (iv) Hsp72 endo-complex Run1. **B:** Hsc70: (i) Hsc70 apo Run2, (ii) Hsc70-SANC00132 Run2, (iii) Hsc70 endo-apo Run2 (iv) Hsc70 endo-complex Run2.

Table S7: Per residue contribution to the total binding free energy. Residues contributing > +/-5 $kJmol^{-1}$ of the total binding free energy were summarised below.

Protein-ligand complex		Per residue contribution to total BFE		Standard deviation
		Residue	Binding energy ($kJmol^{-1}$)	
Hsp72-SANC00132 complex	Run1	ALA392<br>PRO393<br>ILE415<br>GLU439<br>ARG504	-9.2929<br>-5.6193<br>-8.5837<br>9.6769<br>-5.7884	0.8923083
	Run2	VAL391<br>LEU396<br>ILE415<br>GLU439<br>ILE475	-5.2542<br>-11.3954<br>-8.3079<br>-5.9290<br>-5.8271	0.8634035
Hsp72 endo-complex	Run1	ALA392<br>ILE415	-7.0066<br>-9.7801	
	Run2	ARG166<br>ASP390<br>VAL391<br>ALA392<br>LEU394<br>LEU396<br>THR414<br>GLU439<br>LEU481	7.0044<br>10.5007<br>-6.7432<br>-6.6763<br>-6.2393<br>-10.4549<br>-5.7496<br>6.9388<br>-6.0443	1.041394
Hsc70-SANC00132 complex	Run1	Val391<br>ASP501<br>ARG504<br>LYS507	-8.4813<br>-6.7608<br>10.7052<br>14.0571	0.977574
	Run2	PRO393<br>ILE415	-9.4854<br>-7.7102	0.6952183
Hsc70 endo-complex run1	Run1	HIS18<br>GLY19<br>LEU386<br>VAL391<br>GLU439<br>GLY479<br>ARG504	-8.9954<br>-5.0711<br>-5.1133<br>-11.1632<br>-5.1557<br>-5.4216<br>9.8270	0.9081865
	Run2	THR392<br>GLY479<br>ARG504<br>GLU439	-10.0733<br>-6.1143<br>-9.7011<br>9.2959	0.8594879

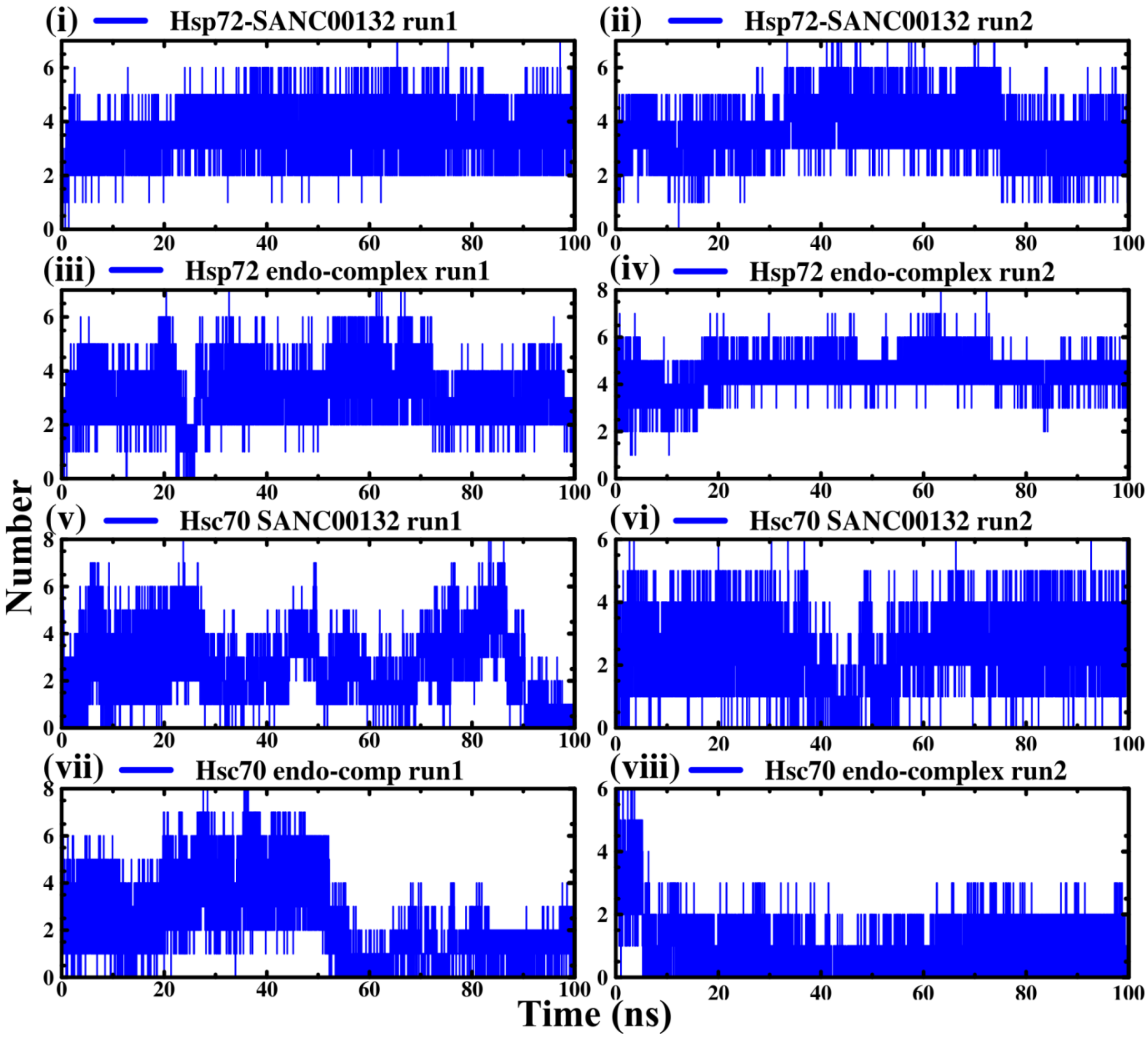

Figure S6: Plots showing the number of hydrogen bonds formed between protein-ligand complexes throughout the simulation period.

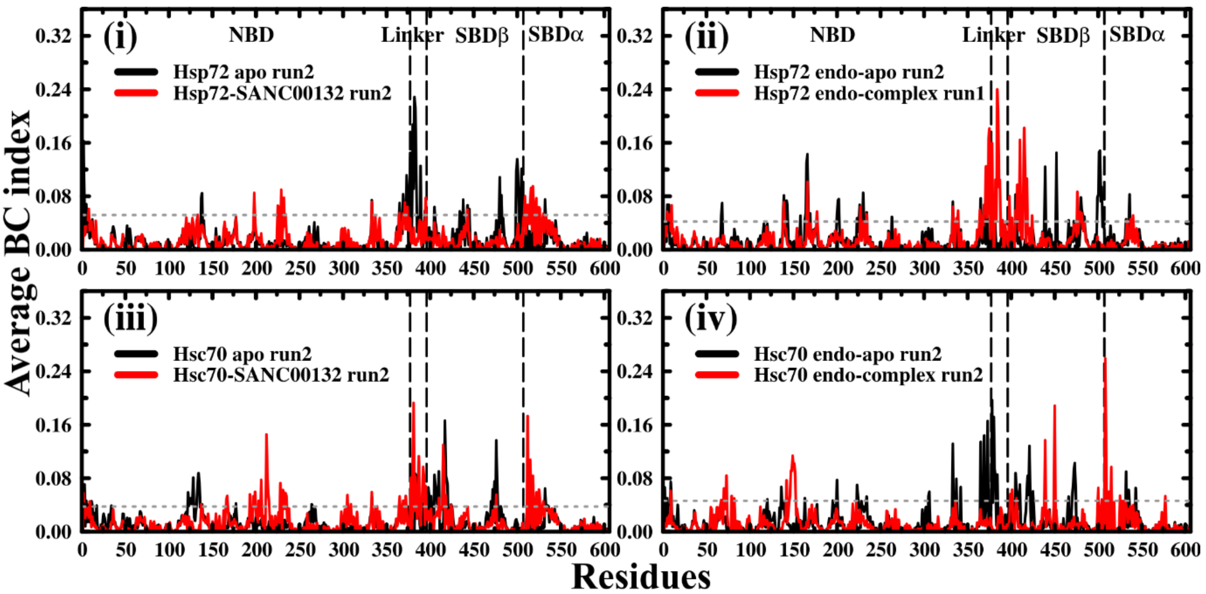

Figure S7: Contact network analysis: Betweenness centrality results for duplicate simulations. The lower threshold values of 0.05 (Hsp72 apo run2), 0.04 (Hsp72 endo-apo run2), 0.04 (Hsc70 apo run2), and 0.05 (Hsc70 endo-apo run2) are indicated by the dotted grey lines.

Table S8: Tabulated summary of residues having low average (L) values. Equivalent residue numbering from complete Hsp72 and Hsc70 sequences (accession numbers NP_005337.2 and AAK17898.1 respectively). **(A and B)** Inhibitor free and inhibitor-bound protein residues translated from **Table 1**.

(A) Inhibitor-free proteins

Protein	Residues
Hsp72	ALA6-THR14, CYY17 - GLN19, GLU27 – ILE29, TYR41, ARG72, LEU124, THR125, GLU132, THR140, ALA142-TYR149, GLN156, LYS159, ASP160, VAL163, ASN168-LEU185, PHE198-LEU200, LEU336, GLY338, ASN364, ASP366, ALA368-GLN389
Hsc70	ALA6-GLN22, GLU27, ILE29, ALA30, PRO39, TYR41, ARG72, GLY75, SER121, MET122, LEU124, THR125, MET127-GLU129, ALA131, GLU132, THR140-ASN151, LYS159-THR163, GLY166-TYR183, GLY338, ALA368-GLY382, GLN389

(B) Inhibitor-bound proteins

Protein	Residues
Hsp72	GLY8-THR13, CYS17-VAL20, GLU25, ILE29, VAL133-TYR149, ILE172-ARG187, LEU196–LEU200, ILE209-THR211, GLU218, LEU334-GLY339, ARG342, LYS361, ASN364-ILE379, MET381, ASP395, ILE420-LYS423, ASP481, ASN483-ILE485
Hsc70	ALA6-THR14, CYS17-PHE21, ILE29, TYR41, LYS71, ARG72, ARG76, SER121, LEU124, THR125, LYS128, GLU129, GLU132, ALA142-TYR149, ASN151, ARG155, GLU156, LYS159, ASP160, GLY162, THR163, GLY166, ASN168-TYR183, PHE198, LEU200, ILE209, THR211, LYS221, ALA368-ILE379, SER381, GLY382, LEU391

Table S9: Residues that yielded high betweenness values: Corresponding residue numbering from complete Hsp72 and Hsc70 sequences (accession numbers NP_005337.2 and AAK17898.1 respectively). (A and B) Residues from inhibitor-free proteins and inhibitor-bound proteins translated from **Table 3**.

(A) Inhibitor-free proteins

Protein	Residues
Hsp72	ILE7, ASP10, GLY12, VAL143, ILE144, LEU170, ARG171, ASN174, ALA182, ASP206, GLU231, ASN235, ASN239, GLY338, ARG342, ALA370, ALA374, ALA377-ILE379, SER381-ASP395, LEU399, SER400, THR410, THR411, TYR443, GLU444, GLU446, ALA448, GLY457, ILE485, ASN487, THR489, THR504-LYS507, ARG509-LEU510, GLU516, SER537, ASN540, ALA541
Hsc70	VAL7, GLY8, ASP10-GLY12, THR14, CYS17, TYR41, LYS88, TRP90, LYS102, PRO116, SER121, LEU124, THR125, MET127, LYS128, ALA133, VAL139, ASN141, VAL143-VAL146, ILE164, ASN174, PRO176, ALA182, LYS188, LEU200, PHE205, LEU228, ASP234, ASN235, ASN239, ALA270, GLY338, ARG342, GLN347, VAL369, ALA370, ALA374, GLN376-ALA378, ASP383, SER385-GLU386, VAL388-LEU392, ILE403, GLU404, ALA406, MET410-VAL412, ILE414, LYS415, THR422-GLN426, THR429, THR430, MET449, GLY459, GLY470, VAL471, GLU475, THR477-ASP479, ASP481, ALA482, LEU486-SER489, THR504-ASP506, ARG509, SER542, ASN540, SER541,TYR545, ASN548, MET549, ASP582

(B) Inhibitor-bound proteins

Protein	Residues
Hsp72	ASP10-CYS17, LEU124, THR125, GLU132, VAL143, ILE144, VAL146, ARG171, ASN174, ALA179-TYR183, LEU196, GLY203, GLU231, ASP234, ASN235, VAL238, ASN239, LEU336, GLY338, ARG342, PRO344, GLN347, VAL369, ALA370, GLY372, ALA374 - LEU380, GLY382, ASP383, ASN387, GLN389-LEU392, ASP395, SER400, LEU403, ALA406, MET410-LEU413, LYS415, THR419-PRO421, LYS423, THR429, THR429, GLN441, ALA448, THR479, ASP481, GLY484, ILE485, LEU486, VAL488, THR489, THR504, ASP506, LYS512, GLU516, GLN520, GLU523, ALA527, SER537, ALA538, ALA541, LEU542, TYR545, MET549
Hsc70	ASP10, GLY12, THR14, TYR15, VAL18, GLU27 - ILE29, ARG72, ARG76, LEU124, THR125, VAL143 - PRO147, PHE150, GLN154 - GLN156, ASN168, VAL169, ARG171, ASN174, PRO176, LEU200, GLY202, THR204, ASP206, THR211, PHE217, GLU218, GLY229, PHE233, ARG236, ASN239, PHE310, GLY338, ARG342, VAL369, ALA370, GLY372, ALA374, ALA377, ASP383, GLU386, VAL388, ASP390, LEU391, LEU392, ASP395 - PRO398, SER400, ILE403, GLU404, ALA406, MET410 - VAL412, LYS415, THR419 - PRO421, GLN441, GLU444, ALA448, LEU455, ASN487, ASN505, LYS507, SER511 - ASP514, GLU516, ARG517, GLN520, GLU523, GLU530, SER537, SER538, ASN540 - TYR545, MET549, ASP582

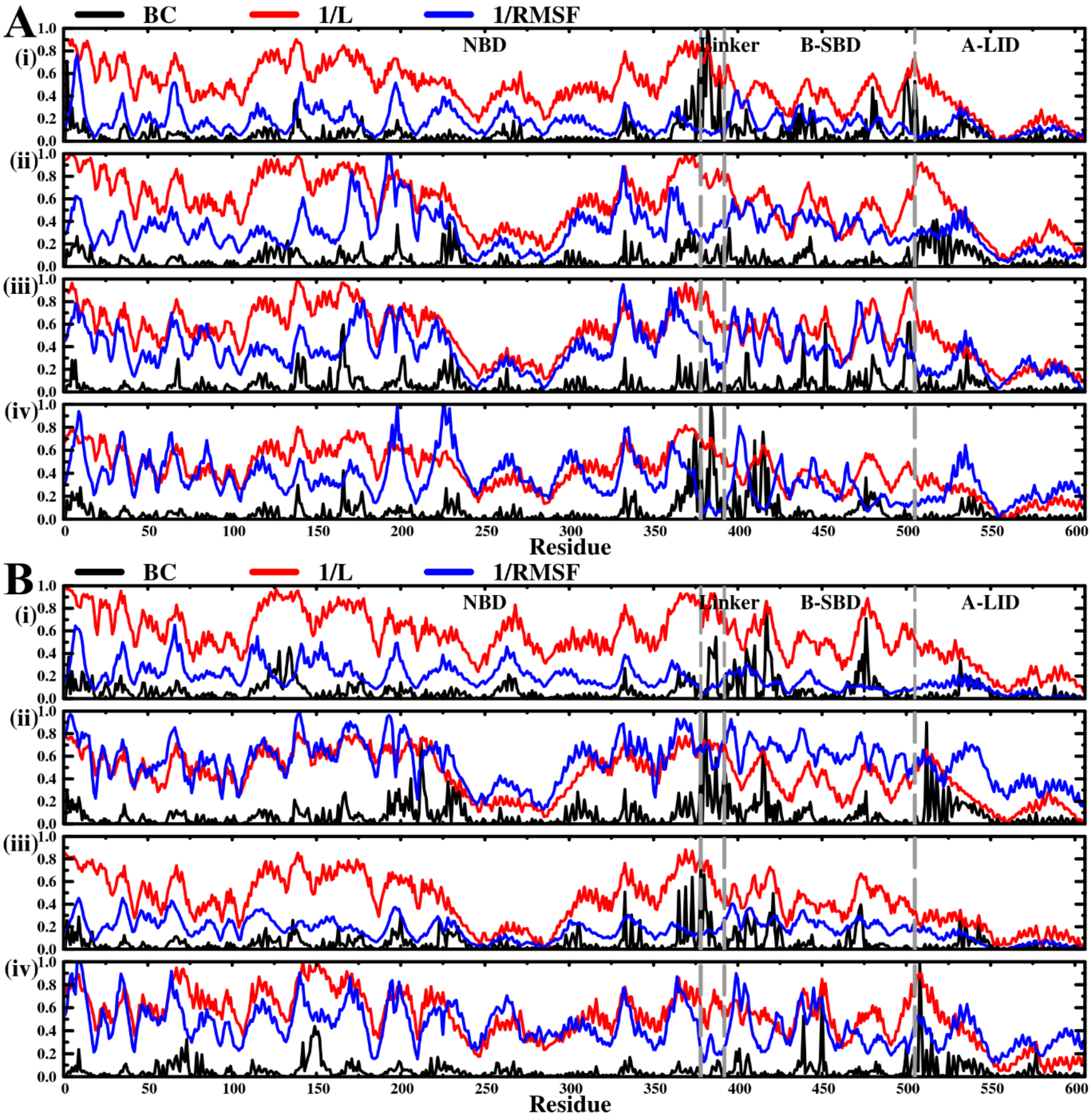

Figure S8: A graphical illustration of correlation between BC, 1/L and 1/RMSF of duplicate simulation results. A: (i) Hsp72 apo run2, (ii) Hsp72-SANC00132 complex run2, (iii) Hsp72 endo-apo run2, (iv) Hsp72 endo-complex run1, **B**: (i) Hsc70 apo run2, (ii) Hsc70-SANC00132 complex run2, (iii) Hsc70 endo-apo run2, (iv) Hsc70 endo-complex run2.

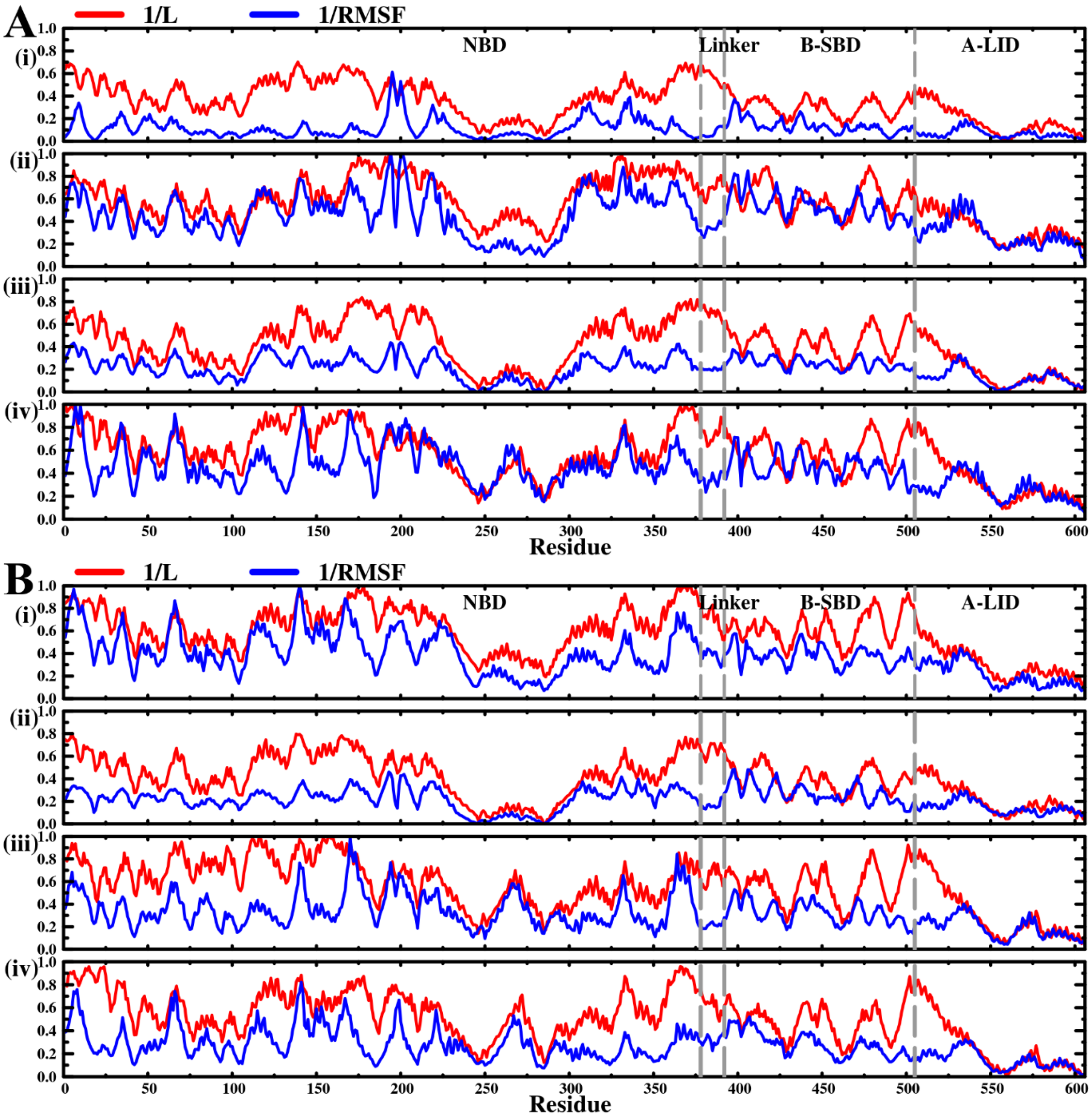
A
1/L
1/RMSF
(i)
(ii)
(iii)
(iv)
NBD
Linker
B-SBD
A-LID
Residue
B
1/L
1/RMSF
(i)
(ii)
(iii)
(iv)
NBD
Linker
B-SBD
A-LID
Residue

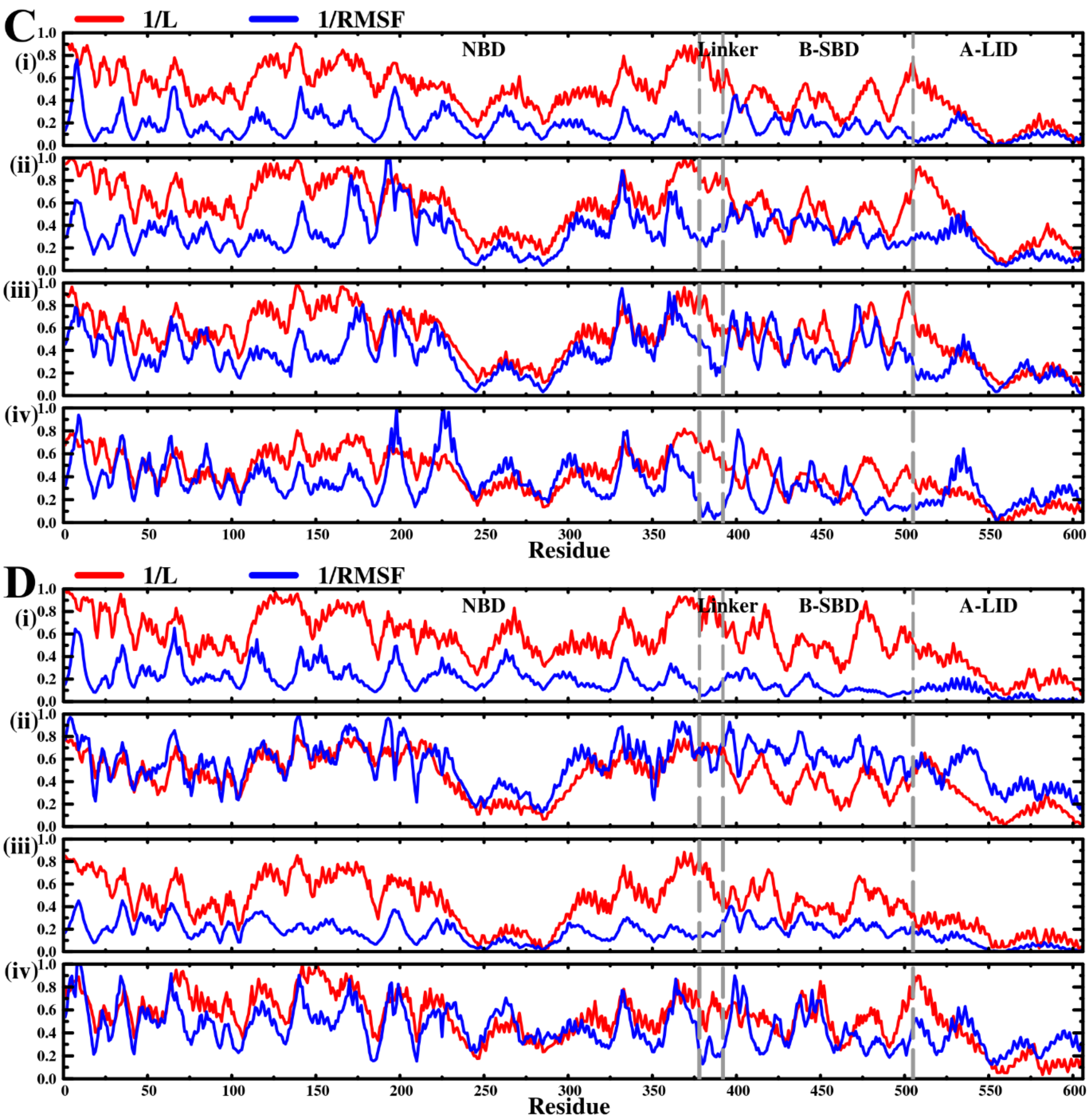

Figure S9: Pairwise comparison of correlation between 1/L and 1/RMSF: A: (i) Hsp72 apo run1, (ii) Hsp72-SANC00132 complex run1, (iii) Hsp72 endo-apo run1, (iv) Hsp72 endo-complex run2, B: (i) Hsc70 apo run1, (ii) Hsc70-SANC00132 complex run1, (iii) Hsc70 endo-apo run1, (iv) Hsc70 endo-complex run1. C: (i) Hsp72 apo run2, (ii) Hsp72-SANC00132 complex run2, (iii) Hsp72 endo-apo run2, (iv) Hsp72 endo-complex run1, D: (i) Hsc70 apo run2, (ii) Hsc70-SANC00132 complex run2, (iii) Hsc70 endo-apo run2, (iv) Hsc70 endo-complex run2.

	NBD (Nucleotide Binding Domain)					SBD (Substrate Binding Domain)				
**Protein**	**L vs RMSF (100ns)**	**L vs RMSF (last 15ns)**	**L^{-1} vs BC**	**$RMSF^{-1}$ (100ns) vs BC**	**$RMSF^{-1}$ (last 15ns) vs BC**	**L vs RMSF (100ns)**	**L vs RMSF (last 15ns)**	**L^{-1} vs BC**	**$RMSF^{-1}$ (100ns) vs BC**	**$RMSF^{-1}$ (last 15ns) vs BC**
**Hsp72 apo Run1**	0.52	0.45	0.45	0.18	0.17	0.48	0.62	0.45	0.45	0.41
**Hsp72 apo Run2**	0.53	0.45	0.58	0.19	0.26	0.67	0.58	0.51	0.21	0.23
**Hsp72-SANC00132 Run1**	0.51	0.83	0.58	0.18	0.35	0.11	0.78	0.56	0.26	0.46
**Hsp72-SANC00132 Run2**	0.61	0.74	0.54	0.13	0.31	0.57	0.78	0.61	0.26	0.26
**Hsp72 endo-apo Run1**	0.54	0.85	0.46	0.17	0.33	0.64	0.79	0.46	0.39	0.34
**Hsp72 endo-apoRun2**	0.67	0.83	0.48	0.25	0.31	0.49	0.72	0.55	0.47	0.29
**Hsp72 endo-complex Run1**	0.59	0.39	0.56	0.38	0.28	0.50	0.14	0.56	0.53	0.00
**Hsp72 endo-complex Run2**	0.62	0.61	0.56	0.22	0.34	0.50	0.68	0.55	0.19	0.34
**Hsc70 apo Run1**	0.45	0.84	0.56	0.12	0.43	0.65	0.88	0.54	0.16	0.43
**Hsc70 apo Run2**	0.42	0.41	0.70	0.12	0.23	0.80	0.72	0.55	0.41	0.37
**Hsc70-SANC00132 Run1**	0.88	0.88	0.40	0.34	0.33	0.54	0.68	0.45	0.40	0.39
**Hsc70-SANC00132 Run2**	0.82	0.82	0.43	0.30	0.39	0.53	0.73	0.54	0.06	0.31
**Hsc70 endo-apo Run1**	0.57	0.59	0.55	0.10	0.38	0.74	0.76	0.53	0.24	0.29
**Hsc70 endo-apo Run2**	0.68	0.76	0.42	0.27	0.29	0.85	0.90	0.52	0.50	0.41
**Hsc70 endo-complex Run1**	0.45	0.64	0.54	0.11	0.27	0.73	0.80	0.51	0.35	0.24
**Hsc70 endo-complex Run2**	0.50	0.59	0.62	0.08	0.38	0.31	0.53	0.46	0.13	0.36

Table S10: Tabulated summary of Pearson's correlation coefficient values calculated domain-wise. The following values were recorded on average: **NBD**: Average L vs RMSF (100ns): r = 0.59, L vs RMSF (last 15ns): r=0.67, L^{-1} vs BC: r =0.53, $RMSF^{-1}$ (100ns) vs BC: r =0.20, $RMSF^{-1}$ (last 15ns) vs BC: r = 0.32. **SBD:** Average L vs RMSF (100ns): r = 0.57, L vs RMSF (last 15ns): r = 0.69, L^{-1} vs BC: r = 0.52, $RMSF^{-1}$ (100ns) vs BC: r = 0.31, $RMSF^{-1}$ (last 15ns) vs BC: r =0.32.

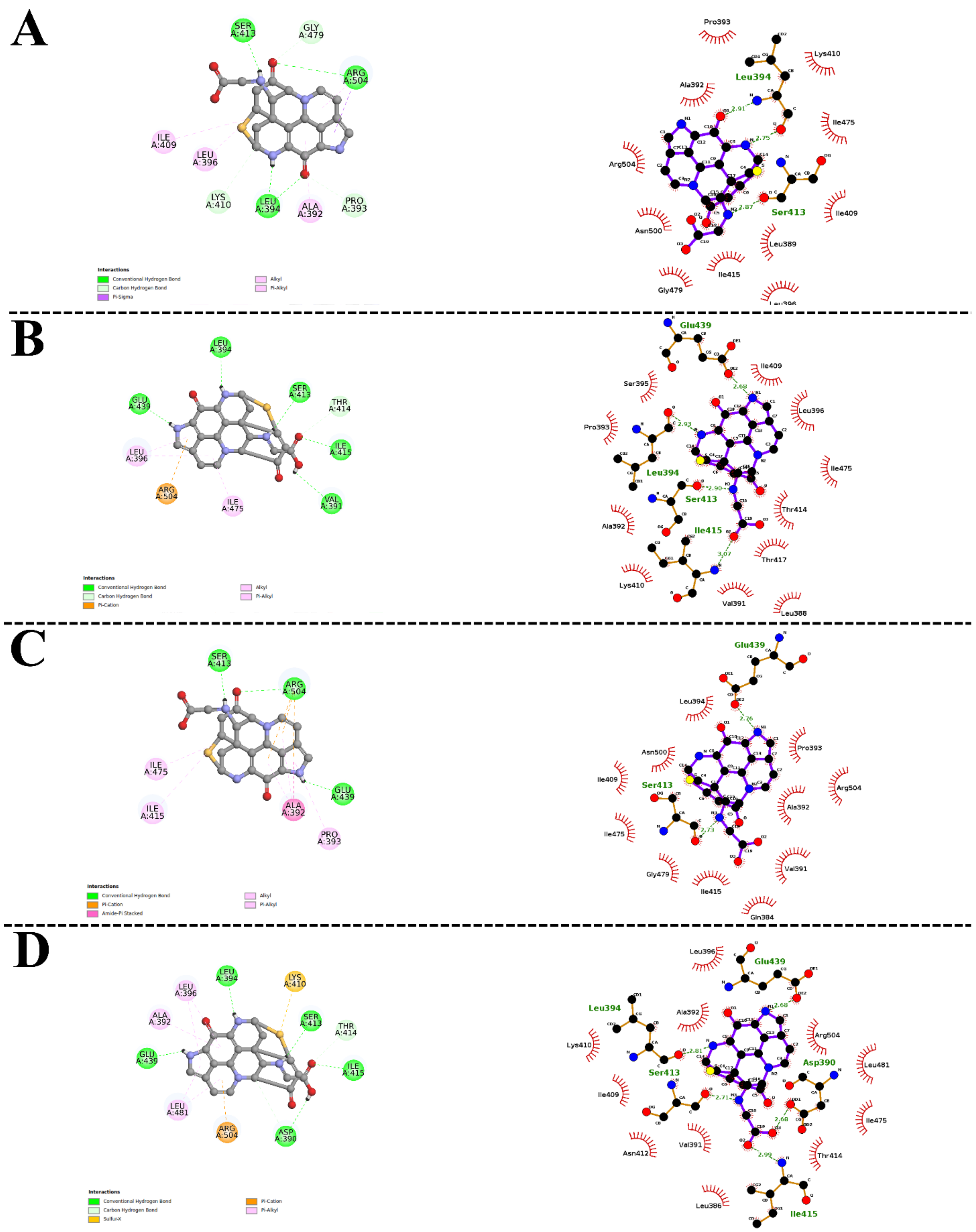

Figure S10: Post MD simulation results: Hsp72-SANC00132 molecular interactions visualised using Discovery studio and LigPlot+. (A) Hsp72-SANC00132 run1, (B) Hsp72-SANC00132 run2, (C) Hsp72 endo-complex run1, (D) Hsp72 endo-complex run2.

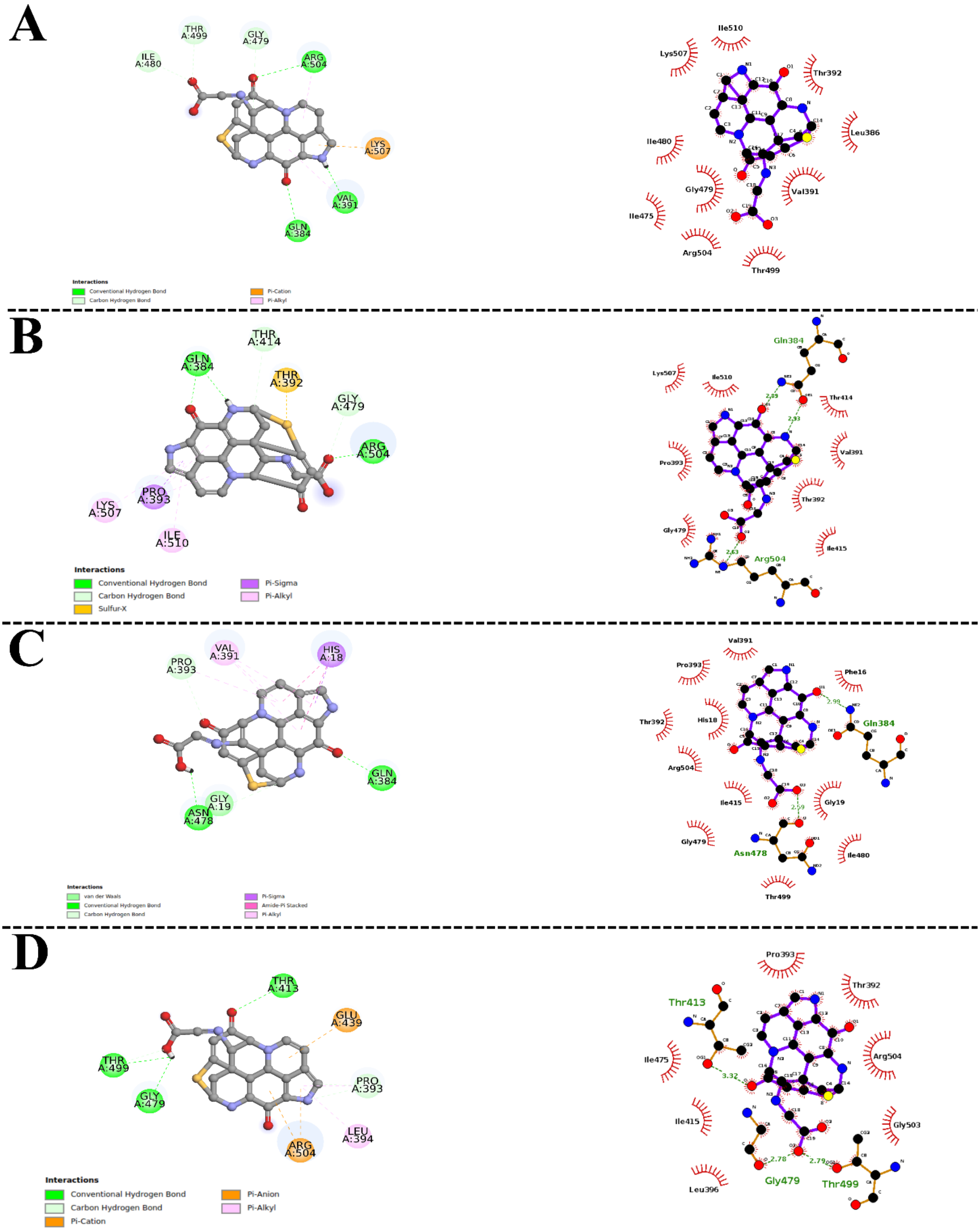

Figure S11: Post MD simulation results. Hsc70-SANC00132 molecular interactions visualised by Discovery studio and LigPlot+. (A) Hsc70-SANC00132 complex run1, (B) Hsc70-SANC00132 complex run2, (C) Hsc70 endo-complex run1, (D) Hsc70 endo-complex run2.

Table S11: Tabulated summary of Hsp72 and Hsc70 residues interacting with SANC00132. Residues matching putative allosteric hotspots identified from BC, L and DnaK were noted. Equivalent amino acid numbering based on the full-length protein sequence was also noted.

Protein	Interacting residues	Putative allosteric residue match (L) Yes = match No = no match	Putative allosteric residue match (BC) Yes = match No = no match	Corresponding putative allosteric residue match (in DnaK)	Full-length sequence equivalent
Hsp72	ALA392	-	Yes	THR395 [1]	ALA397
	PRO393	-	Yes	PRO396 [2]	PRO398
	LEU394	-	-	LEU397 [2,3]	LEU399
	LEU396	-	-	LEU393 [2]	LEU401
	ILE409	-	-	ILE412 [2]	ILE414
	LYS410	-	-	ALA413 [2]	LYS415
	SER413	-	-	-	SER418
	ILE415	-	-	-	ILE420
	ILE475	-	-	ILE478 [2]	ILE480
	ARG504	Yes	-	-	ARG509
Hsc70	GLN384	Yes	Yes	-	GLN389
	VAL391	-	-	VAL394 [1,3]	VAL396
	THR392	-	-	-	THR397
	PRO393	-	-	PRO396 [2]	PRO398
	ILE415	-	Yes	-	ILE420
	GLY479	Yes	Yes	-	GLY484
	ILE480	Yes	-	-	ILE485
	THR499	-	-	THR502 [2]	THR504
	ARG504	-	-	-	ARG504

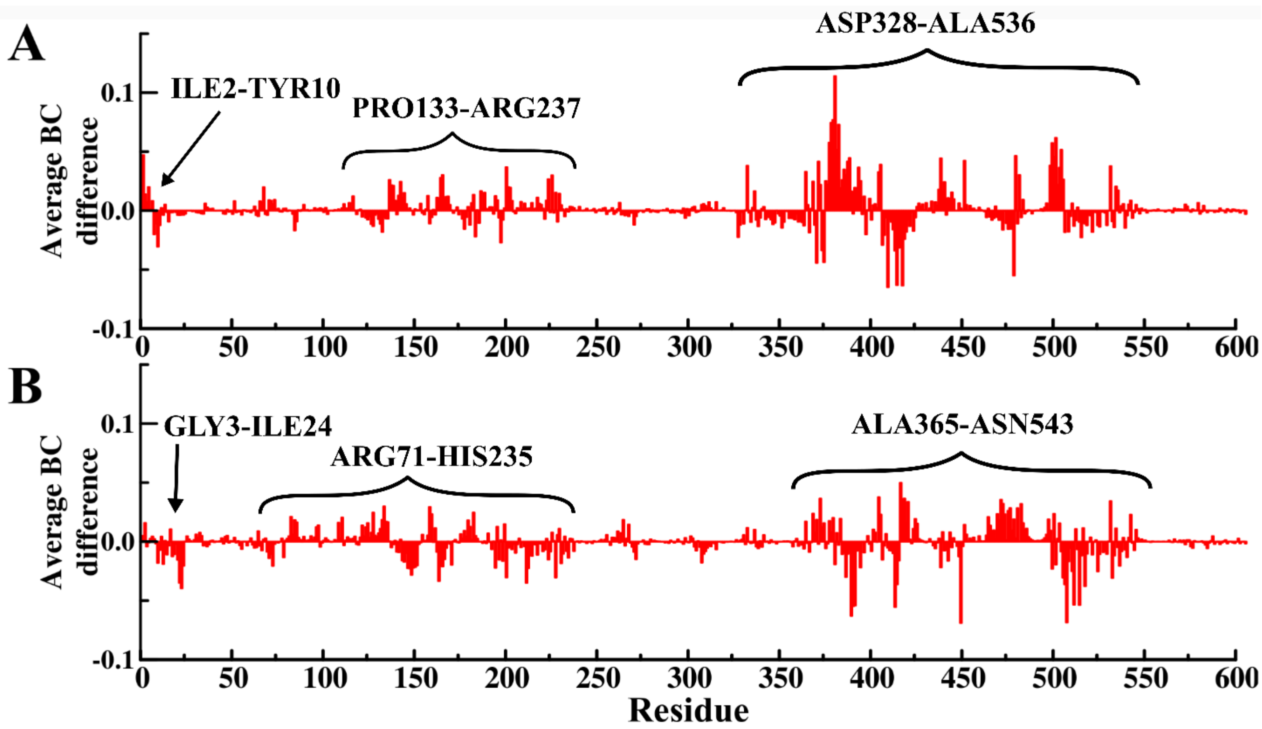

Figure S12: Bar plots displaying protein regions that yielded significant changes in average betweenness centrality values because of ligand binding. Values were obtained by calculating SANC00132-free less SANC00132-bound residue centrality on average. A: Hsp72, B: Hsc70.

Table S12: Tabulated results of residues showing significant changes in average betweenness centrality. Residue numbering is according to *E. coli* DnaK sequence (UniProtKB ID: P0A6Y8). Residues were identified using cutoff values of twice the standard deviation.

Protein	Change	Residues
Hsp72	Positive change (Decreased residue centrality)	ILE2, ARG166, ALA365, ALA372, ASP378, LYS379, SER380, GLU381, ASN382, VAL383, LEU386, LEU387, LEU388, LEU389, LEU394, MET405, THR406, GLU439, GLY452, ILE480, ASN482, THR499, ASN500, ASP501, LYS502, ARG504, LEU505, SER532
	Negative change (Increased residue centrality)	TYR10, GLN371, ILE374, LEU375, LYS410, THR414, ILE415, PRO416, LYS418, GLY479
Hsc70	Positive change (Decreased residue centrality)	VAL134, ILE159, ALA373, MET405, THR417, GLN419, GLN421, THR472, PHE473, ASP476, ALA477, LEU481, VAL483, SER532
	Negative change (Increased residue centrality)	GLU22, ILE23, GLN149, VAL164, ASP201, PHE212, PHE228, LEU387, ASP390, VAL391, THR392, THR414, ILE415, LEU450, SER506, LYS507, GLU508, ARG512, GLN515, GLU518, SER533

Video S1: An accelerated video of 100ns simulation of Hsp72 apo (run1): The protein is represented as a tube.

Video S2: An accelerated video of 100ns simulation of Hsp72-SANC00132 complex (run1): The protein is represented as a tube, whereas SANC00132 is depicted in licorice representation.

Video S3: An accelerated video of 100ns simulation of Hsp72 endo-apo run1 (run1): The protein is represented as a tube, whereas ADP (red) and peptide substrate (green) are shown by licorice representation.

Video S4: An accelerated video of 100ns simulation of Hsp72 endo-complex (run2): The protein is represented as a tube, whereas ADP (red), SANC00132 (blue), and peptide substrate (green) are shown by licorice representation.

REFERENCE

1. English, C.A.; Sherman, W.; Meng, W.; Gierasch, L.M. The Hsp70 interdomain linker is a dynamic switch that enables allosteric communication between two structured domains. *J. Biol. Chem.* **2017**, *292*, 14765–14774, doi:10.1074/jbc.M117.789313.

2. Penkler, D.; Sensoy, Ö.; Atilgan, C.; Tastan Bishop, Ö. Perturbation-Response Scanning Reveals Key Residues for Allosteric Control in Hsp70. *J. Chem. Inf. Model.* **2017**, *57*, 1359–1374, doi:10.1021/acs.jcim.6b00775.

3. Qi, R.; Sarbeng, E.B.; Liu, Q.; Le, K.Q.; Xu, X.; Xu, H.; Yang, J.; Wong, J.L.; Vorvis, C.; Hendrickson, W.A.; et al. Allosteric opening of the polypeptide-binding site when an Hsp70 binds ATP. *Nat. Struct. Mol. Biol.* **2013**, *20*, 900–7, doi:10.1038/nsmb.2583.
